# Two-dimensional turbulence above topography: Vortices and potential vorticity homogenization

**DOI:** 10.1073/pnas.2308018120

**Published:** 2023-10-23

**Authors:** Lia Siegelman, William R. Young

**Affiliations:** ^a^Scripps Institution of Oceanography, University of California, San Diego, CA 92093

**Keywords:** 2D turbulence, vortex, potential vorticity, ocean topography

## Abstract

The evolution of unforced and weakly damped two-dimensional turbulence over random rough topography presents two extreme states. If the initial kinetic energy E is sufficiently high, then the topography is a weak perturbation, and evolution is determined by the spontaneous formation and mutual interaction of coherent axisymmetric vortices. High-energy vortices roam throughout the domain and mix the background potential vorticity (PV) to homogeneity, i.e., in the region between vortices, which is most of the domain, the relative vorticity largely cancels the topographic PV. If E is low, then vortices still form but they soon become locked to topographic features: Anticyclones sit above topographic depressions and cyclones above elevated regions. In the low-energy case, with topographically locked vortices, the background PV retains some spatial variation. We develop a unified framework of topographic turbulence spanning these two extreme states of low and high energy. A main organizing concept is that PV homogenization demands a particular kinetic energy level $E_{♯}$. $E_{♯}$ is the separator between high-energy evolution and low-energy evolution.

## Significance

Two-dimensional (2D) turbulence above topography is a basic model of large-scale ocean flow. This “topographic turbulence” problem has been studied since the seventies. Although vortices are a dominant feature of flat-bottom 2D turbulence, vortices are remarkable mainly by their absence in early work on topographic turbulence. This issue is brought into focus by ocean observations showing that long-lived vortices sit astride prominent topographic features. Using a suite of numerical experiments, we illustrate the phenomenology of topographic turbulence. In the low-energy regime, topography vortices are immobile and locked to topography: Cyclones sit above elevations and anticyclones above depressions. In the high-energy regime, vortices roam freely throughout the domain. We identify the energy scale that separates these regimes.

Seabed topography steers ocean geostrophic turbulence and results in spatial correlations between topography and flow. It is not surprising that topography makes geostrophic turbulence more predictable than evolution above a featureless flat bottom, e.g., Taylor columns are an early example of topographic flow organization. Theories of “topographic turbulence” include the minimum enstrophy hypothesis (1) and predictions based on statistical mechanics (2–4). But a successful and unified framework of topographic turbulence has not emerged in the wake of refs. 1–3.

Theories of topographic turbulence (1–3) were developed before the importance of vortices in flat-bottom two-dimensional turbulence (2DT) was appreciated (5–12). Vortices are a dominant feature of 2DT and also of baroclinic turbulence (13, 14). Although 2DT is a limit of topographic turbulence, statistical theories (1–4) say nothing about how this vortex-dominated limit might be recovered as a special case.

A theory of topographic turbulence must feature vortices and identify the parameters controlling the transition to vortex-dominated 2DT. In this work, we make some preliminary steps in this program. Using numerical experiments, we illustrate the phenomenology of topographic turbulence. We find that some elements of the minimum enstrophy hypothesis proposed by Bretherton and Haidvogel (1), allied with PV homogenization arguments (15), provide useful guidance in explaining the results of these numerical experiments.

The simplest model of topographic turbulence is unforced two-dimensional flow in a rapidly rotating homogeneous fluid layer with uneven depth. If the Rossby number and the fractional change in layer depth are both small, then the quasi-geostrophic approximation (4) applies and the geostrophic velocity is derived from a streamfunction ψ(x,y,t) such that $\left(u,v\right)=\left(-\psi_{y},\psi_{x}\right)$. Material conservation of potential vorticity (PV hereafter) is

[1] $$q_{t}+\psi_{x}q_{y}-\psi_{y}q_{x}=D\zeta \;.$$

In Eq. **1** the PV is

[2] $$q\overset{\text{def}}{=}\zeta +\eta \;,$$

where the relative vorticity is $\zeta \left(x,y,t\right)\overset{\;\text{def}}{=}\psi_{\mathrm{xx}}+\psi_{\mathrm{yy}}$ and η(x,y) is the “topographic PV” (4).

**Fig. 1.**
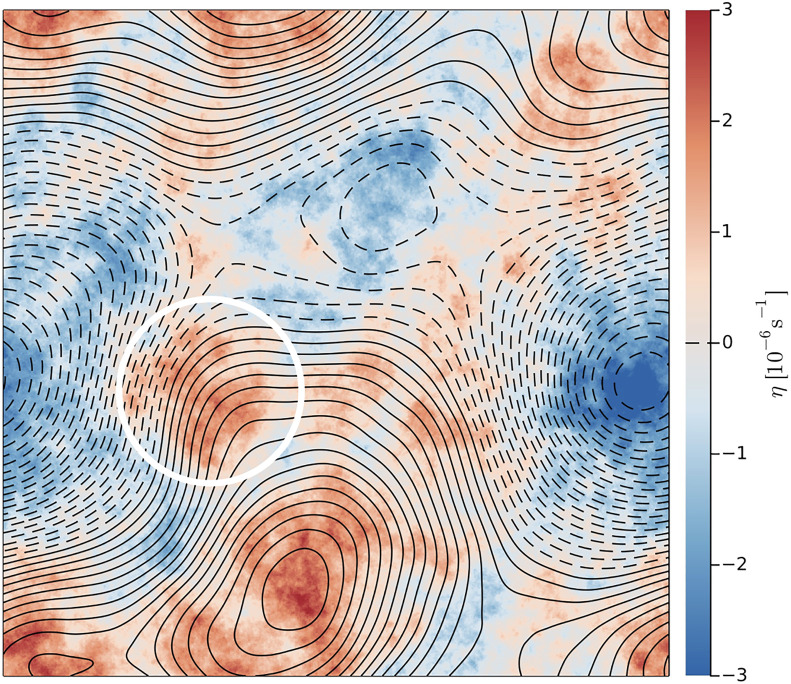
Color shows a topographic PV, η(x,y), with an isotropic $k^{-2}$ spectrum (1). We use this single realization of the topography throughout this work. The contours show $\psi_{♯}\left(x,y\right)$ (solid is positive and dashed is negative) defined via solution of Eq. **9**. The blue regions ($\eta =-f_{0}h_{1}/h_{0}<0$) are topographic depressions ($h_{1}>0$). The white circle encloses a region of elevated topography which is not expressed as a maximum in the low-pass filtered field $\psi_{♯}$. The significance of this region emerges in Section 3.

If the depth is $h_{0}+h_{1}\left(x,y\right)$, with $h_{0}\gg h_{1}\left(x,y\right)$, then $\eta =-f_{0}h_{1}/h_{0}$. Here, $f_{0}$ is the local Coriolis parameter and the big constant $h_{0}$ is an average depth. The topography in Fig. 1 is a single realization with a $k^{-2}$ spectrum. We refer to this as “rough” topography because the topographic-slope spectrum is white.

The model in Eqs. **1** and **2** can be solved pseudospectrally as an unforced initial value problem in an L×L doubly periodic domain. On the right of Eq. **1**, Dζ represents dissipative processes responsible for removal of fine-scale vorticity. The dissipative operator D is implemented by applying a spectral filter to ζ at each time step (Section 7).

Statistical theories (1–3) robustly predict that

[3] $$\langle \zeta \eta \rangle <0,$$

where 〈〉 denotes an area average over the L×L periodic domain. The sign in Eq. **3** is consistent with a thought experiment (4) in which a flow begins with random initial conditions and no initial correlation between ζ and η. Consider a control region defined by a closed curve encircling the peak of a seamount, i.e., a local maximum in η. After evolution from the random initial condition, some fluid within the control region will have arrived from outside points. This new fluid originated from regions with smaller values of η. Because ζ+η is materially conserved the new fluid, once inside the control region, has ζ<0. Thus, importing new fluid to the region above a seamount (η>0) induces anticyclonic (ζ<0) circulation. Likewise, material advection of new fluid into the region above a topographic depression (η<0) induces cyclonic circulation (ζ>0).

Solodoch et al. (16) have recently drawn attention to an interesting apparent failure of Eq. **3** when compared with ocean observations. In the ocean, there is an association between quasi-permanent anticyclonic vortices and bowl-shaped topographic depressions. Striking examples, such as the Mann Eddy (17, 18) and the Lofoten Eddy (19, 20), are anticyclonic vortices (ζ<0) astride topographic depressions (water deeper than $h_{0}$ so that η<0). In contrast to Eq. **3**, and to the thought experiment above, these ocean observations suggest that $\langle \zeta \eta \rangle >0$.

Numerical solutions provided by Solodoch et al. (16) show that the association between anticyclonic vortices and topographic depressions forms spontaneously in much of the parameter space. In these solutions, anticyclones migrate down-slope, collecting at the bottom of the bowl where they merge to form a single, large anticyclone. On the other hand, cyclones climb upslope and out of the bowl (21). Topographically guided segregation of cyclones from anticyclones must be an important process in topographic turbulence.

Vortex segregation also rationalizes the maintenance of bowl-trapped ocean anticyclones by repeated mergers with continuously injected smaller anticyclones from adjacent slope currents, e.g., refs. 19 and 22. These vortex mergers amount to “negative diffusion” or “unmixing” of PV, i.e., anticyclones (ζ<0) move toward minima of the topographic PV (η<0) and thus reinforce the PV minima. Reinforcement of PV extrema by vortex migration, and segregation of cyclones from anticyclones, is antagonistic to PV homogenization (15).

The variation of the Coriolis parameter in the vicinity of planetary poles is dynamically analogous to axisymmetric topographic variations. This results in the formation of either a polar cyclone or multi-cyclone vortex crystals located around the poles (23–26). The poles are analogous to a topographic elevation (η>0), and vortex segregation results in polar accumulation of cyclones (ζ>0). Polar vortex crystals also suggest a ζ-η correlation with the opposite sign to Eq. **3**.

The sign in Eq. **3** correctly characterizes the results of numerical experiments reported below. The various contrary indications summarized above involve strong but spatially localized vortices. Such vortices emerge spontaneously in all our numerical experiments. But the sign in Eq. **3** is determined by the low-level ζ in the background space between these vortices. In Eq. **11**, we introduce another flow-topography correlation that is sensitive to vortex positions. This alternative correlation has the sign suggested by vortex segregation.

## 1. The Minimum Enstrophy Hypothesis (MEH)

If D=0 in Eq. **1**, then both the total enstrophy,

[4] $$Q\overset{\;\text{def}}{=}\frac{1}{2}\langle q^{2}\rangle \;,$$

and energy,

[5] $$E\overset{\;\text{def}}{=}\frac{1}{2}\langle {\left|\nabla \psi \right|}^{2}\rangle \;,$$

are constant in time. With small non-zero D, Q inexorably decreases while E is conserved to a very good approximation. The decrease in Q is a result of the enstrophy cascade to the small length scales on which D is effective. The two-dimensional inverse cascade of energy ensures that E is concentrated on large length scales where D is ineffective.

The minimum enstrophy hypothesis (MEH) — also known as the selective decay hypothesis—of Bretherton and Haidvogel (1) is that enstrophy transfer to small scales and removal by D makes the system Eqs. **1** and **2** evolve toward a state that minimizes Q for specified E.

The associated minimum-enstrophy variational problem results in the Euler–Lagrange equation

[6] $$\underset{\zeta_{⋆}}{\underbrace{\left(\partial_{x}^{2}+\partial_{y}^{2}\right)\psi_{⋆}}}+\eta =\mu \psi_{⋆}\;.$$

The Lagrange multiplier μ(E) in Eq. **6** is determined so that the energy of the minimum enstrophy solution $\psi_{⋆}\left(x,y,\mu \right)$ is equal to E. Eq. **6** also emerges as the relation between the ensemble-averaged PV and the ensemble-averaged streamfunction in some statistical-mechanical theories of flow over topography (2, 3, 27).

**Fig. 2.**
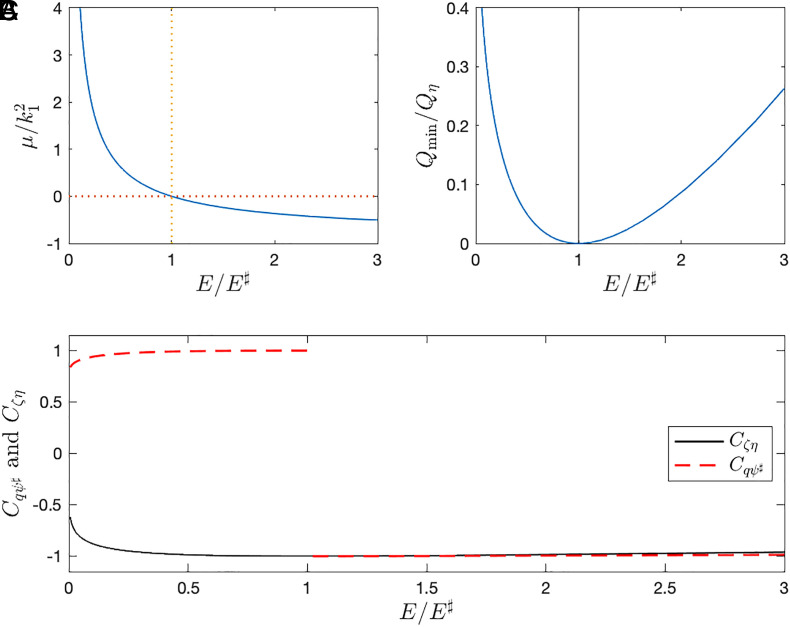
Summary of the minimum enstrophy solution. Panel *A* shows the non-dimensional Lagrange multiplier $\mu /k_{1}^{2}$ as a function of the non-dimensional energy, $E/E_{♯}$. ($k_{1}=2\pi /L$ is the fundamental wavenumber of the L×L doubly periodic domain.) Panel *B* shows the minimum enstrophy as a function of $E/E_{♯}$. On the ordinate in panel *B*, the minimum enstrophy $Q_{\min}$ is normalized with $Q_{\eta }=\left\langle \eta^{2}/2\right\rangle$. Panel *C* shows the correlations $C_{\zeta \eta }\left(\zeta_{⋆}\right)$ in and $C_{q\psi_{♯}}\left(\zeta_{⋆}\right)$. Results above are obtained by solving Eq. **6** using η(x,y) shown in Fig. 1 (see *SI Appendix* for details).

The solution of the variational problem Eq. **6** with η(x,y) in Fig. 1 is summarized in Fig. 2 (*SI Appendix* for some details of the solution). In Fig. 2*A*

[7] $$-k_{1}^{2}<\mu \left(E\right)<\infty ,$$

where $k_{1}=2\pi /L$ is the fundamental wavenumber of the L×L domain. With μ in the range Eq. **7**, the solution of Eq. **6** is a nonlinearly stable solution of the D=0 version of Eq. **1**. Stability follows from the hydrodynamic stability theorems of Arnold (28, 29); see also refs. 3 and 30.

(The variational problem Eq. **6** also has solutions with μ outside the range in Eq. **7**: See figure 1 of Carnevale and Frederiksen (3). These additional solution branches, which are separated by values of μ corresponding to eigenvalues of the Laplacian operator, correspond to unstable solutions with non-extremal enstrophy. Only the main solution branch shown in Fig. 2*A* is relevant to results reported below.)

To systematically discuss the strength of the ζ-η correlation in Eq. **3**, we introduce the normalized correlation

[8] $$C_{\zeta \eta }\left(\zeta \right)\overset{\;\text{def}}{=}\langle \zeta \eta \rangle /\sqrt{\langle \zeta^{2}\rangle \langle \eta^{2}\rangle }\;.$$

Because of the energy constraint, the minimum correlation, $C_{\zeta \eta }=-1$, can only be achieved with homogeneous PV, i.e., μ=0 in Eq. **6** implying that ζ=−η everywhere. But in Fig. 2*C* the correlation $C_{\zeta \eta }\left(\zeta_{⋆}\right)$ is close to the minimum −1 over almost the entire range of energies. Evidently $C_{\zeta \eta }\left(\zeta_{⋆}\right)\approx -1$ is efficient at minimizing Q.

### Homogeneous PV.

The special case μ=0 in Eq. **6** is homogeneous PV (15). In this special case, the streamfunction $\psi_{♯}\left(x,y\right)=\psi_{⋆}\left(x,y,0\right)$ is determined by solution of

[9] $$\left(\partial_{x}^{2}+\partial_{y}^{2}\right)\psi_{♯}+\eta =0\;.$$

The contours in Fig. 1 show $\psi_{♯}$. The energy of the homogenized solution,

[10] $$E_{♯}\overset{\;\text{def}}{=}\frac{1}{2}\langle |\nabla \psi_{♯}{|}^{2}\rangle \;,$$

is used to define a convenient non-dimensional energy $E/E_{♯}$, e.g., the abscissas in Fig. 2. We refer to $E/E_{♯}<1$ (μ>0) as the low-energy branch and $E/E_{♯}>1$ (μ<0) as the high-energy branch.

A main conclusion of this work is that the non-dimensional parameter $E/E_{♯}$ controls the transition between topographically dominated turbulence ($E/E_{♯}\ll 1$) and flat-bottom turbulence ($E/E_{♯}\gg 1$). Numerical results in Sections 2 and 3 identify $E/E_{♯}=1$ as a critical value.

The homogenized solution suggests another measure of the correlation between vorticity and topography:

[11] $$C_{q\psi_{♯}}\left(q\right)\overset{\;\text{def}}{=}\langle q\psi_{♯}\rangle /\sqrt{\langle q^{2}\rangle \langle {\psi_{♯}}^{2}\rangle }\;.$$

The sign of $C_{q\psi_{♯}}\left(\zeta_{⋆}+\eta \right)$ is extremely sensitive to the energy level: in Fig. 2*C*, $C_{q\psi_{♯}}\left(\zeta_{⋆}+\eta \right)$ jumps discontinuously from +1 to −1 at $E/E_{♯}=1$. To explain the discontinuity, if E is close to $E_{♯}$, then |μ|≪1. In this case, the approximate solution of the Euler–Lagrange equation [**6**] is $q\approx \mu \psi_{♯}$. This approximation can be used to evaluate the averages in Eq. **11**, e.g., $\langle q\psi_{♯}\rangle \approx \mu \langle \psi_{♯}^{2}\rangle$. One quickly finds that $C_{q\psi_{♯}}=\mu /\left|\mu \right|$.

### Topographic Advection.

The streamfunction $\psi_{♯}\left(x,y\right)$ defined by Eq. **9** is the basis of a transformation of Eqs. **1** and **2**. Define ϕ(x,y,t) by

[12] $$\psi \left(x,y,t\right)=\psi_{♯}\left(x,y\right)+\phi \left(x,y,t\right)\;.$$

In terms of ϕ the PV is

[13] $$q=\left(\partial_{x}^{2}+\partial_{y}^{2}\right)\phi \;.$$

Material conservation of PV is

[14] $$q_{t}+\underset{\;\text{TA}}{\underbrace{{\psi_{♯}}_{x}q_{y}-{\psi_{♯}}_{y}q_{x}}}+\underset{\;\text{VA}}{\underbrace{\phi_{x}q_{y}-\phi_{y}q_{x}}}=D\zeta \;.$$

In Eq. **14**, TA is “topographic advection” and VA is “vortex advection.”

Eq. **14** is an exact restatement of Eq. **2** and by itself produces no additional information or simplification. If, however, the flow evolves to produce vortices moving through a background of uniform PV (as in numerical solutions reported below) then Eq. **14** justifies viewing $\psi_{♯}$ as a streamfunction associated with the topography. The $k^{-2}$-topography in Fig. 1 has extrema on all resolved scales. Not all of these tiny bumps and dips in the topography will affect the motion of strong vortices. The streamfunction $\psi_{♯}$ in Fig. 1 is a low-pass filtered version of η that reveals large-scale topography. TA is the advection of vortices by a background flow with homogeneous PV. This background flow has the negative ζ-η correlation anticipated in Eq. **3**. VA is the remote effect on a vortex produced by the irrotational velocity induced by other distant vortices.

## 2. Case Study $E/E_{♯}=1$

We begin by discussing a numerical solution with $E/E_{♯}=1$. The initial condition is a random monoscale relative vorticity ζ(x,y,0). Monoscale means that ζ(x,y,0) is characterized by a single well-defined length scale $L_{\mathrm{init}}$. Parameter values are summarized in Table 1 and further details are in Section 7.

**Table 1.** Key parameter values used throughout the solution suite

Symbol	Description	Numerical value
L	Domain size L×L	$10^{6}$ m
$n_{x}\times n_{y}$	Resolution	1,024 × 1,024
dt	Time step	1,500s
nsteps	Number of integration steps	$10^{6}$
$t_{\mathrm{final}}$	The final time $1.5\times 10^{9}\;\;\text{s}$	47.53 y
$L_{\mathrm{init}}$	Length scale of initial ζ	$2\pi \times 10^{4}$ m
$k_{1}$	Fundamental wavenumber	2π/L
$\eta_{\mathrm{rms}}$	Root mean square of η	$10^{-6}\;\;{\text{s}}^{-1}$
$Q_{\eta }$	Topographic enstrophy $\langle \eta^{2}/2\rangle$	$5\times 10^{-13}\;\;{\text{s}}^{-2}$
$E_{♯}$	$\langle |\nabla \psi_{♯}{|}^{2}\rangle /2$	$5\times 10^{-3}\;\;{\text{m}}^{2}\;\;{\text{s}}^{-2}$
$1/k_{1}\sqrt{E_{♯}}$	Dynamical time scale	26 days
$U_{♯}$	Velocity $\sqrt{2E_{♯}}$	$10^{-1}\;m\;\;{\text{s}}^{-1}$

According to the MEH, $E/E_{♯}=1$ is the separator between high- and low-energy solutions. At $E/E_{♯}=1$, the MEH predicts that ζ+η=0, $Q_{\min}=0$ and $C_{\zeta \eta }=-1$. We test these three predictions against a numerical solution.

**Fig. 3.**
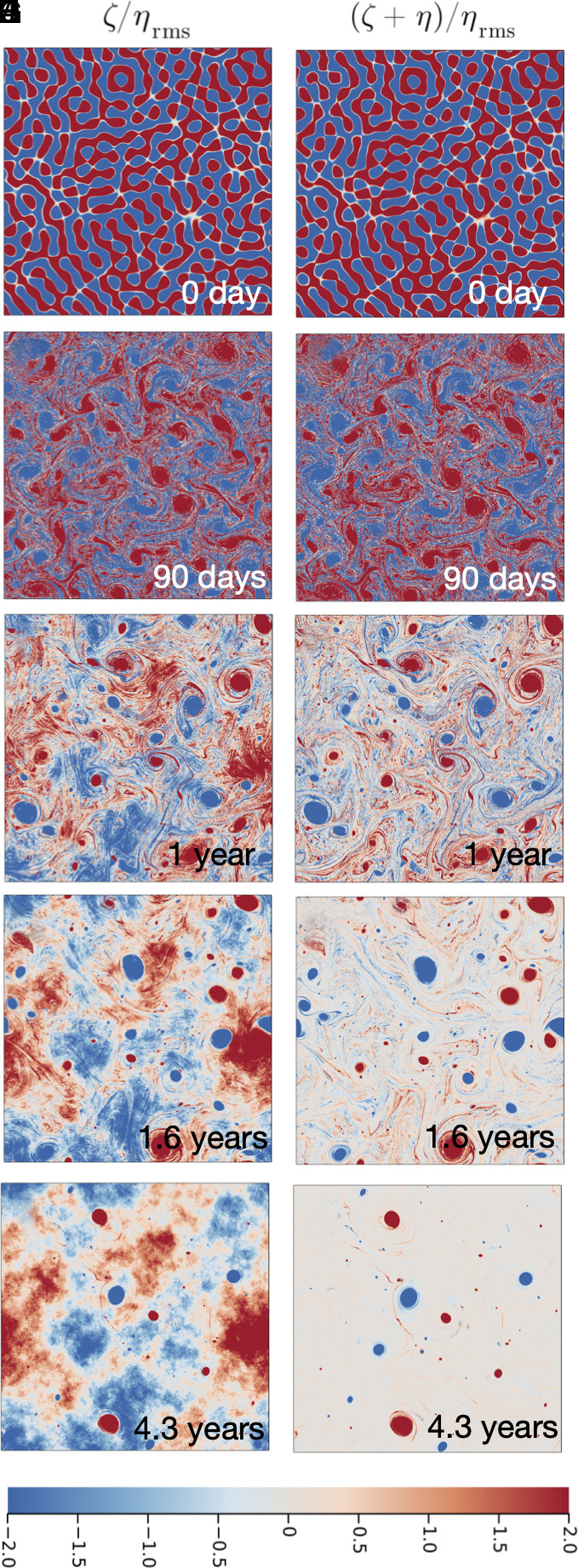
Time evolution of the run with $E/E_{♯}=1$. Initial relative vorticity ζ is a random monoscale field with $L_{\mathrm{init}}=2\pi \times 10$ km. The *Left* column (panels *A*, *C*, *E*, *G*, and *I*) shows the relative vorticity ζ and *Right* (panels *B*, *D*, *F*, *H*, and *J*) shows the potential vorticity ζ+η. The background PV is well homogenized at the final time t≈4.3 y in panels *I* and *J*. Results of a longer run to t=47.53 y are shown in Fig. 6 *C* and *G*.

Fig. 3 shows snapshots of the solution (see *SI Appendix* for an animation). In Fig. 3*A* and *B* ζ≫η and so that ζ is indistinguishable from ζ+η. One year of evolution (Fig. 3*E* and *F*) results in:

(i) emergence of a vortex gas familiar from studies (5–12) of plain and simple 2DT;
(ii) PV mixing, resulting in partial cancellation between ζ and η and production of a “background flow” in the region between vortices.

Subsequent evolution, shown in Fig. 3*G* and *H*, homogenizes the background PV. In Fig. 3*J* the PV consists of nine or ten large vortices and smaller vortex debris moving in a sea of spatially uniform background PV.

It is easy to overlook the relative vorticity ζ of the background flow because it is so much smaller than vortex-core ζ: In Fig. 3*J*, the vortex-core ζ is larger by at least a factor of one hundred than the background ζ. (To make the background ζ visible, the colorbar in Fig. 3 shows only low levels.) Low-level background ζ plays a dominant role in determining global ζ-η correlations such as $C_{\zeta \eta }$.

### Status of the MEH.

The numerical solution in Fig. 3—with $E/E_{♯}=1$—presents both a failure and a success of the MEH. The success is that after some evolution the background PV mixes to homogenity. The failure is that $Q_{\min}=0$ is not achieved.

We dwell on the failure. In terms of

[15] $$Q_{\eta }\overset{\;\text{def}}{=}\frac{1}{2}\langle \eta^{2}\rangle \;,$$

the initial enstrophy is $Q\left(0\right)=108Q_{\eta }$ and in Fig. 3*J* $Q\left(4.3\;\;\text{y}\right)=5.2Q_{\eta }$. Although there has been significant dissipation of enstrophy, Q(4.3y) is well above $Q_{\min}=0$. In Section 3, we evolve this solution to 47.53 y and find $Q\left(47.53\;\;\text{y}\right)=1.8Q_{\eta }$. The enstrophy in excess of the minimum is contained within the vortex cores. The vortex-gas model (9) assumes that vorticity extrema are shielded from the enstrophy cascade. In topographic turbulence, vortex-core shielding also preserves a significant (relative to $Q_{\eta }$) amount of the initial enstrophy.

With $E/E_{♯}=1$, the MEH also predicts that $C_{\zeta \eta }=-1$. The initial correlation, established by the random phases used to generate ζ in Fig. 3*A* is essentially zero. This correlation decreases to $C_{\zeta \eta }\left(4.3\;\;\text{y}\right)=-0.345$ (and further to −0.596 at 47.53 y). If ζ≈−η characterized the entire flow then $C_{\zeta \eta }$ would have to be −1. Instead, because of vortices, $C_{\zeta \eta }$ remains stubbornly larger than −1. This is a partial success of MEH: At least, the sign of $C_{\zeta \eta }$ is correct and the correlation is decreasing toward −1, albeit very slowly as some vortices are eliminated by late-time mergers.

Conclusion: The failures of the MEH result from the spontaneous formation of vortices and preservation of enstrophy within vortex cores.

## 3. Different Energy Levels

We further test the MEH with numerical experiments using sixteen values of the energy level:

[16] $$E/E_{♯}=\left\{0.05,\;0.1,\;0.25,\;0.5,\;0.55,\;0.6,\;0.65,\;0.7,\;0.75,\;0.875,\;1,\;1.125,\;1.25,\;1.5,\;1.75,\;2\right\}.$$

**Fig. 4.**
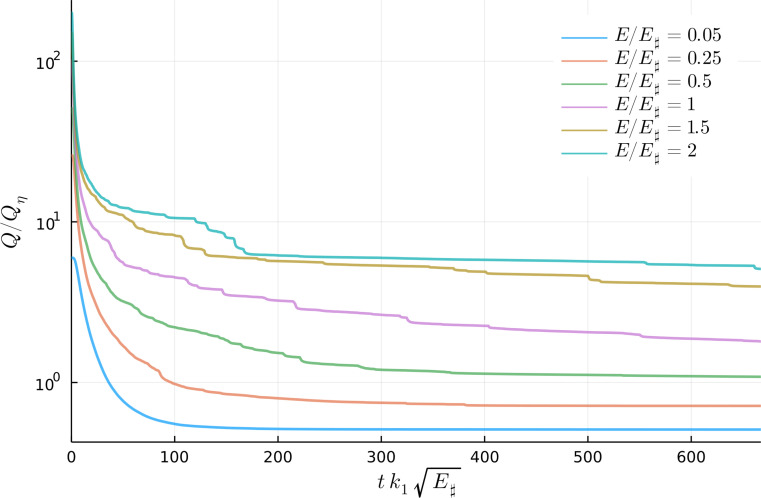
Enstrophy decay for six runs with different energy levels. The final time is $1.5\times 10^{9}$ s, or 47.53 y. The ordinate is non-dimensionalized with $Q_{\eta }$ in Eq. **15**. The initial enstrophy varies linearly with $E/E_{♯}$ and is given by Eq. **27**.

**Fig. 5.**
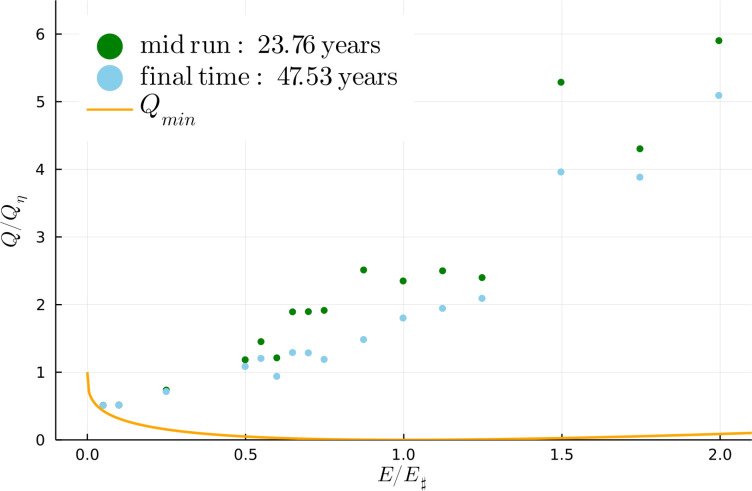
Enstrophy as a function of $E/E_{♯}$ for all 16 runs. The green points show enstrophy at t=23.765 y and the blue points show enstrophy at end of the run with t=47.53 y. The solid curve is the minimum enstrophy $Q_{\min}\left(E/E_{♯}\right)$ obtained by solution of Eq. **6**. For runs with $E/E_{♯}\geq 0.50$, the final enstrophy is greatly in excess of $Q_{\min}$.

Fig. 4 shows the evolution of enstrophy Q(t) for six representative runs. Q(t) decays strongly from its initial value Q(0), while E(t) decreases by less than 1% from E(0) (*SI Appendix*). Loss of Q with constant E is consistent with the MEH. But Fig. 5 shows that unless $E/E_{♯}$ is rather small (e.g., $E/E_{♯}=0.05$), the final enstrophy is not quantitatively predicted by the solution of the variational problem [**6**]. If $E/E_{♯}$ is larger than about 0.5, the final enstrophy in Fig. 5 is greatly in excess of $Q_{\min}$. Except for the runs with the smallest values of $E/E_{♯}$, all points in Fig. 5 fall well above the minimum enstrophy curve provided by the solution of Eq. **6**.

In Fig. 4, there is a fast initial decrease in enstrophy followed by a gradual slow decrease. During the second slow stage, the emergent vortices move through mutual advection and interaction with the background flow. As in 2DT, close chance encounters between like-signed vortices result in merger and expulsion of filaments of vorticity which are then mixed into the background PV. Vortex mergers decrease enstrophy: Some of the abrupt downward steps at long time in Fig. 4 result from individual mergers between large vortices. But in all sixteen solutions, vortices remain even after 47.53 y—hence the excess enstrophy in Fig. 5.

**Fig. 6.**
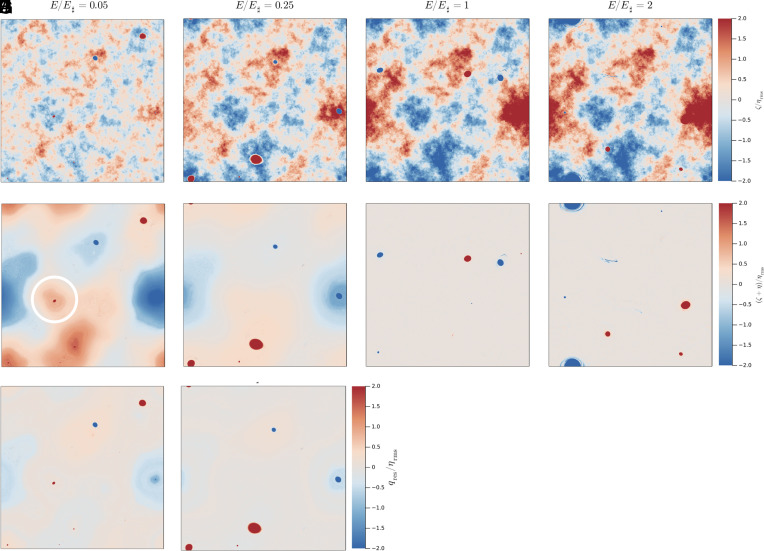
Final (t=47.53 y) states for the four runs with different energies in each column. The upper row (panels *A*, *B*, *C*, and *D*) shows relative vorticity ζ and the middle row (panels *E*, *F*, *G*, and *H*) shows q=ζ+η. Background PV is homogenized in high-energy solutions in panels *G* and *H*. Low-energy solutions in panels *E* and *F* have non-homogeneous background PV. The third row (panels *I* and *J*) shows $q_{\mathrm{res}}$ in Eq. **18**. The high-energy runs in *G* and *H* have $\mu_{\mathrm{emp}}=0$ so that $q_{\mathrm{res}}=q$. To emphasize the background variation of PV, the color range is narrow, e.g., the vortices have $q/\eta_{\mathrm{rms}}$ as large as ±40 and are strongly saturated in this illustration. The white circle in panel *E* encloses the exceptional vortex with a locked position that is not an extrema of $\psi_{♯}$.

Fig. 6 shows the state of four runs after 47.53 y of evolution. The top row shows the relative vorticity ζ and the middle row the PV ζ+η.

### High-Energy Solutions: $E/E_{♯}\geq 1$.

The high-energy runs in Fig. 6*G* and *H* both exhibit impressive background PV homogenization. (Fig. 6*G* is the subsequent development of the state in Fig. 3*J*).

According to the MEH, the runs with $E/E_{♯}>1$ should have non-zero negative μ(E); see Fig. 2*A*. In contrast to this MEH prediction, the six runs with $E/E_{♯}\geq 1$ all develop homogeneous background PV (15). We speculate that total PV homogenization is the result of background PV mixing by roaming vortices (below). Because of PV homogenization, the decomposition Eq. **20** will be useful in understanding high-energy vortex dynamics, e.g., once the PV is homogenized a point-vortex model is useful.

Animations in *SI Appendix* show that vortices in the high-energy runs in Fig. 6*G* and *H* roam throughout the domain. Roaming is episodically interrupted when vortices orbit many times around extrema of the topographic streamfunction $\psi_{♯}\left(x,y\right)$ in Fig. 1. These $\psi_{♯}$-extrema can be interpreted as stable stagnation points of the topographic advection produced by $\psi_{♯}$. $\psi_{♯}$-extrema are stagnation points because the topographic velocity associated with the streamfunction $\psi_{♯}$ vanishes at extrema. Rather than being swept away by the TA associated with saddle points of $\psi_{♯}$, vortices tend to remain close to $\psi_{♯}$-extrema.

Vortices of both signs orbit cyclonically around the minima in $\psi_{♯}$ (topographic depressions) and anticyclonically around the maxima of $\psi_{♯}$ (topographic elevations). Visual impressions based on *SI Appendix* animations do not indicate long-term topographic vortex trapping in these high-energy solutions. Nonetheless, the correlation $C_{\psi_{♯}q}$ is weakly negative indicating that the trajectories of high-energy cyclones (q>0) spend more time over depressions $\psi_{♯}<0$ (Section 5).

### Low-Energy Solutions: $E/E_{♯}<1$.

The low-energy runs in Fig. 6*E* and *F* do not have homogeneous background PV (Section 4). But vortex nucleation occurs at low energy, e.g., even the run with $E/E_{♯}=0.05$ in Fig. 6*E* has three strong vortices. Spontaneous vortex formation and long-term vortex survival are characteristic of all solutions.

Animations in *SI Appendix* show that vortices in the low-energy runs in Fig. 6*E* and *F* are stationary and locked to the extrema of $\psi_{♯}$ in Fig. 1. Low-energy locking binds anticyclones (q and ζ negative) to topographic depressions (η and $\psi_{♯}$ negative). This is the correlation of the Lofoten and Mann eddies. We speculate that ocean conditions correspond to the low-energy branch.

There is an “exceptional vortex” surrounded by the white circle in Fig. 6*E*. (This is the same white circle shown in Fig. 1.) The low-energy run with $E/E_{♯}=0.1$ also exhibits a topographically locked cyclone at this location (see *SI Appendix* for an animation). These exceptional vortices are stationary but there is not an extremum of $\psi_{♯}$ within the white circle. Explaining the location of the exceptional vortices in very low-energy runs motivates a closer look at the departures of the low-energy background PV from homogeneity (Section 4).

### Vortex Mobility: Transition between Roaming and Locking.

The mobility of the emergent vortices depends on $E/E_{♯}$. With $E/E_{♯}\geq 1$ vortices roam throughout the domain with occasional episodes during which a vortex orbits around extrema of $\psi_{♯}$. If $E/E_{♯}$ is decisively less than one (e.g., $E/E_{♯}=0.25$) then vortices become immobile and are locked to extrema of $\psi_{♯}$. At intermediate energy levels, such as $E/E_{♯}=0.75$, vortices endlessly orbit around $\psi_{♯}$-extrema. The transition between high-energy roaming and low-energy locking seems to involve vortices spending increasingly long sojourns orbiting around $\psi_{♯}$-extrema as $E/E_{♯}$ is reduced, e.g., see *SI Appendix* for an animation showing $E/E_{♯}=0.75$. Systematic quantification of the transition between vortex roaming and vortex locking as a function of energy level $E/E_{♯}$ requires development of a vortex-tracking algorithm (or an inspired theory) and is beyond our scope here.

### Effective Diffusivity of a Passive Scalar.

Basile Gallet has noted that transport properties of the flow, quantified by the effective diffusivity of a passive scalar with an imposed uniform gradient (31, 32), should be very different in the two extreme states $E/E_{♯}\ll 1$ and $E/E_{♯}\gg 1$. With $E/E_{♯}\gg 1$, the effective diffusivity results from chaotic mixing by roaming vortices (14). The effective diffusion—in this case a turbulent eddy diffusivity—should depend weakly, if at all, on the explicit (molecular) diffusivity of the passive scalar. In the other limit, with $E/E_{♯}\ll 1$, and vortices locked to topographic stagnation points, the flow is nearly steady. For a steady flow, the effective diffusivity is controlled by molecular diffusion. At intermediate values of $E/E_{♯}$, one might probe the transition between roaming and locking by measuring the dependence of the effective diffusivity on molecular diffusivity.

### Summary.

Vortices spontaneously form at all sixteen values of $E/E_{♯}$ in Eq. **16**. In the high-energy runs, vortices roam throughout the domain. In the low-energy cases, the vortices are locked to $\psi_{♯}$-extrema. Low-energy vortex locking associates anticyclones with topographic depressions and cyclones to topographic elevations.

## 4. An Empirical ψ-q Relation for the Background Flow

The low-energy solutions in Fig. 6*E* and *F* have a background flow with non-uniform PV. Inspection of ψ-q scatter plots (see Section 7 and *SI Appendix*) suggests that the background flow is characterized by an approximate linear relation between q and ψ:

[17] $$\zeta +\eta \approx \mu_{\mathrm{emp}}\psi \;.$$

**Fig. 7.**
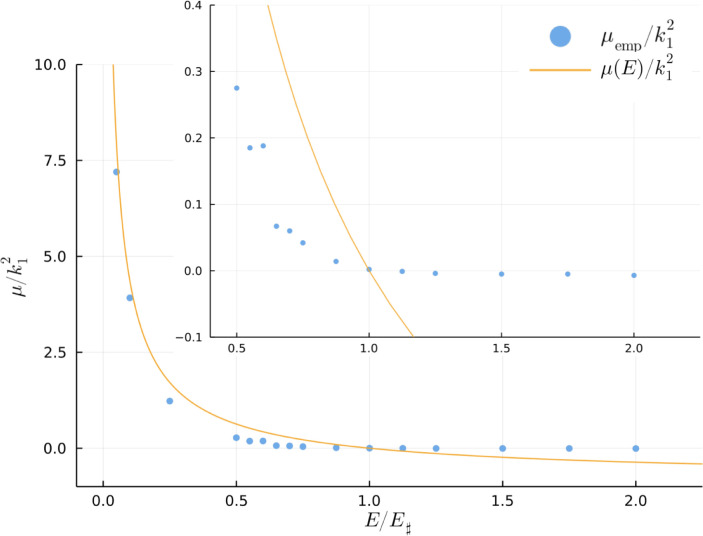
Slope of the empirical ψ-q relation, $\mu_{\mathrm{emp}}/k_{1}^{2}$, as a function of the non-dimensional energy, $E/E_{♯}$. Inset zooms on the region around $E/E_{♯}=1$. The high-energy branch has $\mu_{\mathrm{emp}}$ close to zero, i.e., homogenized background PV.

In Eq. **17**, $\mu_{\mathrm{emp}}\left(E\right)$ is an empirical slope determined by first removing vortex outliers from a ψ-q scatter plot and then applying least-squares fitting to the remaining background points. Fig. 7 summarizes our estimated $\mu_{\mathrm{emp}}\left(E\right)$ for the sixteen solutions. The claim above that the high-energy solutions have homogeneous background PV is substantiated because the six solutions with $E/E_{♯}\geq 1$ have $|\mu_{\mathrm{emp}}|/k_{1}^{2}<0.01$. On the other hand, the low-energy runs in Fig. 7 have $\mu_{\mathrm{emp}}>0$ and $\mu_{\mathrm{emp}}$ increases as $E/E_{♯}$ is reduced. At very low energy in Fig. 7 (e.g., $E/E_{♯}\leq 0.1$) $\mu_{\mathrm{emp}}\left(E\right)$ is close to μ(E) obtained by solution of the variational problem Eq. [**6**]. But generally on the low-energy branch $0<\mu_{\mathrm{emp}}\left(E\right)<\mu \left(E\right)$.

Fig. 6*I* and *J* show the “residual” PV,

[18] $$q_{\mathrm{res}}\overset{\;\text{def}}{=}q-\mu_{\mathrm{emp}}\psi \;,$$

of the low-energy solutions. Much of the variation of background q in Fig. 6*E* and *F* is removed in $q_{\mathrm{res}}$. This confirms that the linear ψ-q relation in Eq. **17** provides at least a rough approximation to the background PV of the low-energy runs. If conjecture Eq. **17** survives further scrutiny then we might be glimpsing a generalized form of the MEH. Our acceptance of Eq. **17** is tentative because ψ-q scatterplots are noisy and $\mu_{\mathrm{emp}}$ has some sensitivity to the threshold used to remove the very large vortex outliers.

We are confident that the six solutions with $E/E_{♯}\geq 1$ have a background flow that is very close to homogeneous PV and that the ten solutions with $E/E_{♯}\leq 0.875$ have a background flow with non-homogeneous PV. $E/E_{♯}=1$ is a critical energy level separating flows which have enough initial energy to mix background PV to homogeneity from weaker flows which retain long-time spatial variation in background PV. This main conclusion is not hostage to Eq. **17**.

### Topographic Advection Again.

In Eqs. **12**–**14** we used the uniform PV streamfunction $\psi_{♯}$ to separate the background flow from the vortex component ϕ. This separation works well for runs with $E/E_{♯}\geq 0.25$. But the very low energy runs ($E/E_{♯}=0.05$ and 0.1) have stronger inhomogeneities in background PV. In this section, we show that the mysterious position of the exceptional vortex in Fig. 6*E* results from applying the inappropriate assumption of homogeneous PV to define the TA streamfunction in these very low energy cases.

We use the empirical relation Eq. **17** to introduce a generalization of the transformation in Eqs. **12**–**14**. With $\mu_{\mathrm{emp}}$ determined from the numerical solution, define $\psi_{\mathrm{emp}}$ as the solution of

[19] $$\left(\partial_{x}^{2}+\partial_{y}^{2}\right)\psi_{\mathrm{emp}}+\eta =\mu_{\mathrm{emp}}\psi_{\mathrm{emp}}\;.$$

On the high-energy branch, where $\mu_{\mathrm{emp}}$ is close to zero, $\psi_{\mathrm{emp}}=\psi_{♯}$. With moderately low energy –$E/E_{♯}=0.5$ – $\psi_{\mathrm{emp}}$ is only slightly different from $\psi_{♯}$.

**Fig. 8.**
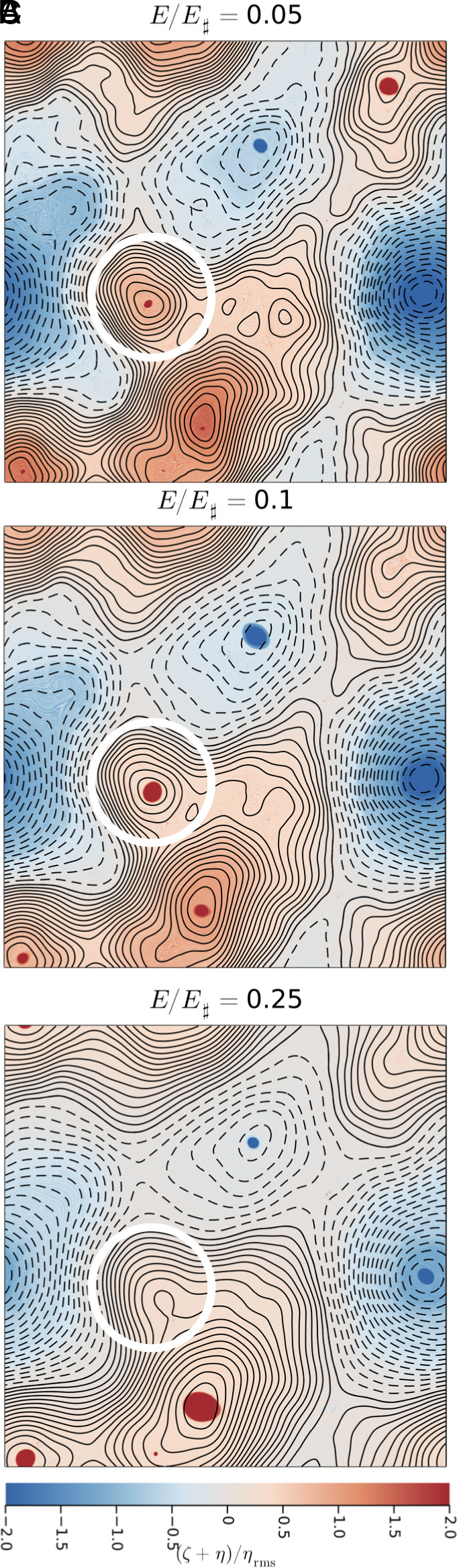
Panels *A*, *B*, and *C* show a topographic PV=ζ+η. The contours show $\psi_{\mathrm{emp}}$ (solid is positive and dashed is negative) defined via solution of Eq. **19**. Panels show different energy levels as indicated. With $E/E_{♯}=\left\{0.05,0.1\right\}$ there is a new (compared to Fig. 1) extremal point inside the white circle. In panels *A* and *B*, cyclones are locked to this $\psi_{\mathrm{emp}}$-extremum.

The filtering defined by Eq. **19** is more spatially local than the inverse Laplacian used to produce $\psi_{♯}$ from η. Thus some topographic features which are eliminated in $\psi_{♯}$ are expressed in $\psi_{\mathrm{emp}}$. For example, in Fig. 8, the streamfunction $\psi_{\mathrm{emp}}$ has an extremal point at the location of the exceptional vortices in the low-energy runs.

Given $\psi_{\mathrm{emp}}$, the vortex component ϕ(x,y,t) is defined by

[20] $$\psi \left(x,y,t\right)=\psi_{\mathrm{emp}}\left(x,y\right)+\phi \left(x,y,t\right)\;.$$

In terms of ϕ, the PV, ζ+η, is

[21] $$q=\left(\partial_{x}^{2}+\partial_{y}^{2}\right)\phi +\mu_{\mathrm{emp}}\psi_{\mathrm{emp}}.$$

Because of the final term, Eq. **21** is more complicated than Eq. **13**. Using these new variables, material conservation of PV in Eq. **1** is

[22] $$q_{t}+\underset{\;\text{TA}}{\underbrace{{\psi_{\mathrm{emp}}}_{x}q_{y}-{\psi_{\mathrm{emp}}}_{y}q_{x}}}+\underset{\;\text{VA}}{\underbrace{\phi_{x}q_{y}-\phi_{y}q_{y}}}=D\zeta \;.$$

Again VA denotes vortex-advection and TA is topographic advection, i.e., advection of PV by the steady streamfunction $\psi_{\mathrm{emp}}\left(x,y\right)$.

The main success of $\psi_{\mathrm{emp}}$ is that in Fig. 8, the exceptional vortices (enclosed by the white circles) are locked to extremal points of $\psi_{\mathrm{emp}}$. This $\psi_{\mathrm{emp}}$-extremum is not present in $\psi_{♯}$.

## 5. Vorticity-Topography Correlations

**Fig. 9.**
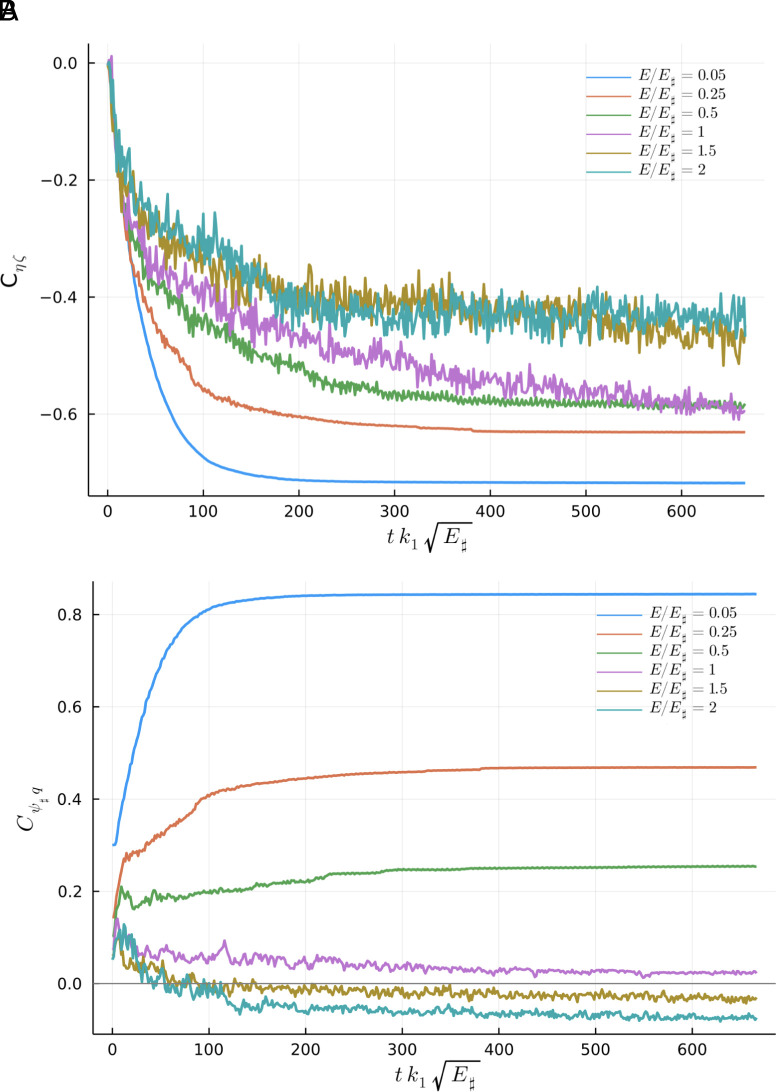
Time series of the vorticity-topography correlation for six runs with different energy levels. (*A*) $C_{\zeta \eta }$. (*B*) $C_{\psi_{♯}q}$.

**Fig. 10.**
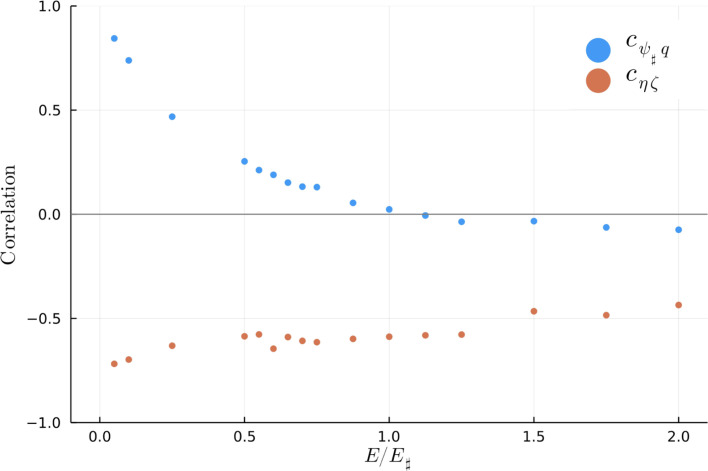
Summary of vorticity-topography correlations averaged over the last $50\;t\;k_{1}\;\sqrt{E_{♯}}$ (corresponding to 3.6 y) in sixteen numerical experiments. $C_{\zeta \eta }$ is negative in all sixteen runs. $C_{q\psi_{♯}}$ changes sign near $E/E_{♯}=1$. On the high-energy branch ($E/E_{♯}>1$), $C_{\psi_{♯}q}$ is weakly negative. On the low energy branch ($E/E_{♯}<1$) $C_{\psi_{♯}q}$ is positive. Positive $C_{\psi_{♯}q}$ is diagnostic of vortex locking with topographic features, e.g., anticyclones (q<0 over depressions$\psi_{♯}<0$).

Fig. 9 shows the evolution of the correlations $C_{\zeta \eta }\left(t\right)$ and $C_{\psi_{♯}q}\left(t\right)$ for six representative solutions. Fig. 10 shows the value of the correlations after 47.53 y of evolution. (Some averaging at the end of the time series in Fig. 9 is used to remove rapid temporal fluctuations in the high-energy runs.)

In Fig. 9*A* $C_{\zeta \eta }\left(0\right)$ is close to zero and immediately becomes negative (this happens in all sixteen runs). $C_{\zeta \eta }\left(t\right)<0$ is in agreement with the introductory thought experiment (4) used to rationalize Eq. **3**. But $C_{\zeta \eta }\left(t\right)$ is not as negative as one might perhaps expect: The high-energy runs ($E/E_{♯}\geq 1$) have homogeneous background PV. If ζ+η≈0 is characteristic of the entire flow then $C_{\zeta \eta }=-1$. Instead, because of vortices, at long times all runs have $C_{\zeta \eta }\left(t\right)$ closer to −0.5 than −1.

The other correlation $C_{\psi_{♯}q}\left(t\right)$ in Figs. 9 and 10 is positive for low-energy solutions ($E/E_{♯}<1$) and weakly negative for high-energy solutions ($E/E_{♯}\geq 1$). $C_{\psi_{♯}q}$ changes sign very close to $E/E_{♯}=1$ in Fig. 10.

The decomposition $\psi =\psi_{♯}+\phi$ in Eq. **12** provides a simple explanation of $\langle q\psi_{♯}\rangle >0$ on the low-energy branch. Calculating $E=\langle {\left|\nabla \psi \right|}^{2}\rangle /2$ one finds

[23] $$E=E_{♯}+\langle \nabla \phi \cdot \nabla \psi_{♯}\rangle +\underset{\;\text{vortex energy}}{\underbrace{\frac{1}{2}\langle {\left|\nabla \phi \right|}^{2}\rangle }}\;.$$

The “cross energy” is $\langle \nabla \phi \cdot \nabla \psi_{♯}\rangle =-\langle q\psi_{♯}\rangle$, and so we can rewrite Eq. **23** as

[24] $$\langle q\psi_{♯}\rangle =E_{♯}-E+\frac{1}{2}\langle {\left|\nabla \phi \right|}^{2}\rangle \;.$$

On the low-energy branch $E_{♯}-E>0$ and it follows from Eq. **24** that $\langle q\psi_{♯}\rangle >0$.

(In *SI Appendix*, we provide a generalization of Eq. **24** using $\left({\text{ψ}}_{emp}\right)\text{:}\;E_{emp}\overset{def}{=}\langle \frac{1}{2}{\left|\nabla {\text{Ψ}}_{emp}\right|}^{2}\rangle$. If $E_{\mathrm{emp}}-E$ is positive, then so are $\langle q\psi_{\mathrm{emp}}\rangle$ and the cross energy $\langle \nabla \psi_{\mathrm{emp}}\cdot \nabla \phi \rangle$. But $\psi_{♯}$ is a well-defined property of the topographic PV η and is independent of the initial energy E. No empirical fitting of ψ-q scatter plots is required to define $\psi_{♯}$. Thus, there are significant advantages in using $\psi_{♯}$ rather than $\psi_{\mathrm{emp}}$.)

The machinations leading to Eq. **24** provide an intuitive explanation for $\langle q\psi_{♯}\rangle >0$ in the low energy case. In these runs, $E_{♯}$ alone exceeds the small energy E provided by the initial condition. The positive-definite vortex energy in Eq. **23** makes the situation even worse. But the decomposition $\psi =\psi_{♯}+\phi$ is not orthogonal in the energy norm: The “cross energy” $\langle \nabla \phi \cdot \nabla \psi_{♯}\rangle$ in Eq. **23** has indefinite sign.

To understand how $\langle \nabla \phi \cdot \nabla \psi_{♯}\rangle$ might be negative, imagine placing a small intense cyclonic vortex at the center of a background anticyclonic circulation. The far-field irrotational azimuthal velocity of the vortex is in opposition to the velocity of the background flow so that the vector sum of the vortex velocity and the background velocity results in partial cancellation. Because of this cancellation, the energy of the superposition is less than the energy of the constituents.

Weakly negative $C_{\psi_{♯}q}$ for high-energy runs in Figs. 9 and 10 indicates that roaming vortices preferentially visit regions in which $\psi_{♯}$ has the opposite sign to the vortex q: anticyclones (q<0) spend more time in regions with anticyclonic background circulation ($\psi_{♯}>0$).

$C_{q\psi_{♯}}$ is the most decisive probe of vortex locking to the large-scale topographic features revealed by $\psi_{♯}$. $C_{\zeta \eta }$ reveals correlations between the background flow and the topographic PV η on all scales. The complementary correlations $C_{\zeta \eta }$ and $C_{q\psi_{♯}}$ have opposite signs on the low-energy branch.

## 6. Discussion and Conclusion

We have focused here on the $k^{-2}$-model topography in Fig. 1. The slope spectrum is white so that this is an idealized model of rough topography. Main results are:

1. Vortices nucleate from random initial conditions —even from low-energy initial conditions;
2. With low initial energy, vortex segregation collects anticyclones above topographic depressions and cyclones above elevated topography;
3. Low-energy topographically trapped vortices are surrounded by a weaker background flow with opposite signed vorticity.

Numerical solutions using smooth regular model topography produce similar results (16). We conclude that the three phenomena above are main features of two-dimensional turbulence above both rough and smooth topography.

Ocean observations of the Lofoten and Mann anticyclones are consistent with these being low-energy configurations as in points 2 and 3 above, e.g., the velocity of the topographically locked anticyclonic vortex is in opposition to the surrounding cyclonic background circulation.

The non-dimensional energy $E/E_{♯}$ is a useful organizing principle. In our suite of numerical experiments, $E/E_{♯}=1$ is the separator between low-energy and high-energy solutions. Flow properties change continuously as we vary $E/E_{♯}$. If $E/E_{♯}\geq 1$, the background PV is homogeneous (15). But if $E/E_{♯}<1$, then the departures from homogeneous background PV, quantified in Fig. 7, are at first small but grow as $E/E_{♯}$ is reduced to a low level.

The minimum enstrophy hypothesis (1) has significant failures. On the high-energy branch, the MEH predicts that μ in the Euler–Lagrange Equation [**6**] is in the range $-k_{1}^{2}<\mu \leq 0$. But numerical solutions show that μ is close to zero for all values of E greater than $E_{♯}$. Vortex nucleation shields significant enstrophy from the cascade to high wavenumbers so that the long-term enstrophy is much greater than the MEH minimum (Fig. 5).

This idealized study ignores the β-effect, drag on the bottom, large-scale mean flow, and forcing. These processes are included in a previous study (33) and the phenomenology of the flow is different e.g. although there was partial PV homogenization, vortices did not spontaneously form. It would also be informative to investigate two-layer baroclinic turbulence over topography. This system can be forced by specifying a large-scale uniform zonal velocity in the top layer. In the flat-bottom case the ensuing baroclinic instability results in spontaneous formation of vortices. The energy level and heat transport of flat-bottom baroclinic turbulence is sensitively controlled by the bottom drag coefficient (13, 14). Perhaps topography alleviates this extreme sensitivity to bottom drag?

We conclude by speculating on the connection between single vortices trapped by topographic turbulence and multi-cyclone vortex crystals located at the Jovian poles (34). The variation of the Coriolis parameter in the vicinity of planetary poles is dynamically analogous to axisymmetric topographic variations. Initial-value experiments, similar to the ones described here, result in the formation of multi-cyclone vortex crystals (26). But in this study we did not find multi-vortex crystal formation above the topography in Fig. 1. Because of vortex segregation in low-energy runs we did, however, observe the collection of same-signed vortices all orbiting close to extrema of $\psi_{♯}$. Because of same-sign vortex merger, these vortex swarms soon condense into a single trapped vortex. The peculiarity of Jovian polar dynamics is that after segregation creates a polar swarm of cyclones, vortex merger does not operate. Instead, the members of the swarm eventually organize into a regularly spaced pattern, usually with a central polar cyclone. We speculate that this orderly arrangement around the pole might be related to conservation of angular momentum in axisymmetric polar geometry. With the irregular topography in Fig. 1, topographic form stress can transfer angular momentum between the vortex swarm and the solid Earth.

## 7. Materials and Methods

### Model Framework.

The barotropic quasi-geostrophic model is based on the GeosphysicalFlows.jl modeling framework (35), using the SingleLayerQG module with an infinite deformation radius and no forcing or dissipation. We take advantage of the GPU functionality (36) provided by GeosphysicalFlows.jl, speeding up the computations by a factor of ∼70 compared to running on 24 CPUs. The computational domain is doubly periodic with size L×L and $k_{1}=2\pi /L$ is the fundamental wavenumber. Our resolution is 1,024 × 1,024. The system is time-stepped forward in Fourier space using a fourth-order Runge–Kutta time stepper, with spectral filtering of the relative vorticity indicated by Dζ in Eq. **1**.

The high-wavenumber filter D is applied to ζ at the end of each time step (35). D is based on the non-dimensional wavenumber $k^{\prime }=k/k_{\max}$, where k=|k| and $k_{\max}=512\times k_{1}$. The filter is

[25] $$\;\text{filter}\left(k\right)=\left\{\begin{array}{cc} 1 & \;k^{\prime }\leq k_{\;\text{cutoff}}^{\prime }\;,\\ \exp\left[-\alpha {\left(k^{\prime }-k_{\;\text{cutoff}}^{\prime }\right)}^{p}\right] & \;k^{\prime }>k_{\;\text{cutoff}}^{\prime }\;, \end{array}\right.$$

with $k_{\;\text{cutoff}}^{\prime }=\frac{2}{3}$ ($k_{\max}^{\prime }=1$ in non-dimensional notation), p=4 and $\alpha =-\log\delta /{\left(1-k_{\;\text{cutoff}}^{\prime }\right)}^{p}$. Given the order p, the coefficient α is computed so that the filter value that corresponds to the highest wavenumber is some small value, δ, taken to be close to machine precision (37). This filter originates from ref. 38 and has been used previously in the quasi-geostrophic systems in refs. 39 and 40. More details can be found https://fourierflows.github.io/FourierFlowsDocumentation/previews/PR353/timestepping/.

### The Initial Condition.

We initialize the computations by specifying a random monoscale relative vorticity ζ(x,y,0). Monoscale means that ζ(x,y,0) is characterized by a single well-defined length scale $L_{\mathrm{init}}$ with corresponding wavenumber $k_{\mathrm{init}}=2\pi /L_{\mathrm{init}}$. We used $k_{\mathrm{init}}/k_{1}=100/2\pi$, where $k_{1}=2\pi /L$ is the fundamental wavenumber of the L×L periodic domain.

The energy E is specified by adjusting the amplitude of the initial relative vorticity ζ(x,y,0). The initial relative enstrophy, $Q_{\zeta }\left(0\right)=\frac{1}{2}\left\langle \zeta {\left(x,y,0\right)}^{2}\right\rangle$, can then be estimated as

[26] $$Q_{\zeta }\left(0\right)\approx k_{\mathrm{init}}^{2}E\;.$$

In non-dimensional variables $Q_{\zeta }\left(0\right)$ is

[27] $$\frac{Q_{\zeta }\left(0\right)}{Q_{\eta }}\approx \underset{=100.3}{\underbrace{{\left(\frac{k_{\mathrm{init}}}{k_{1}}\right)}^{2}\frac{k_{1}^{2}E_{♯}}{Q_{\eta }}}}\;\frac{E}{E_{♯}},$$

where $Q_{\eta }$ is defined in Eq. **15** and the non-dimensional number 100.3 results the $k^{-2}$ topographic spectrum and the numerical value of $E_{♯}$. With Eq. **27** the total initial enstrophy is

[28] $$\frac{Q(0)}{Q_{\eta }}\approx 100.3\;\frac{E}{E_{♯}}+1\;.$$

For most of our runs, the initial total enstrophy above is very much greater than the minimum enstrophy obtained by solution of the variational problem Eq. **6**.

### Derivation of $\mu_{\mathrm{emp}}$.

**Fig. 11.**
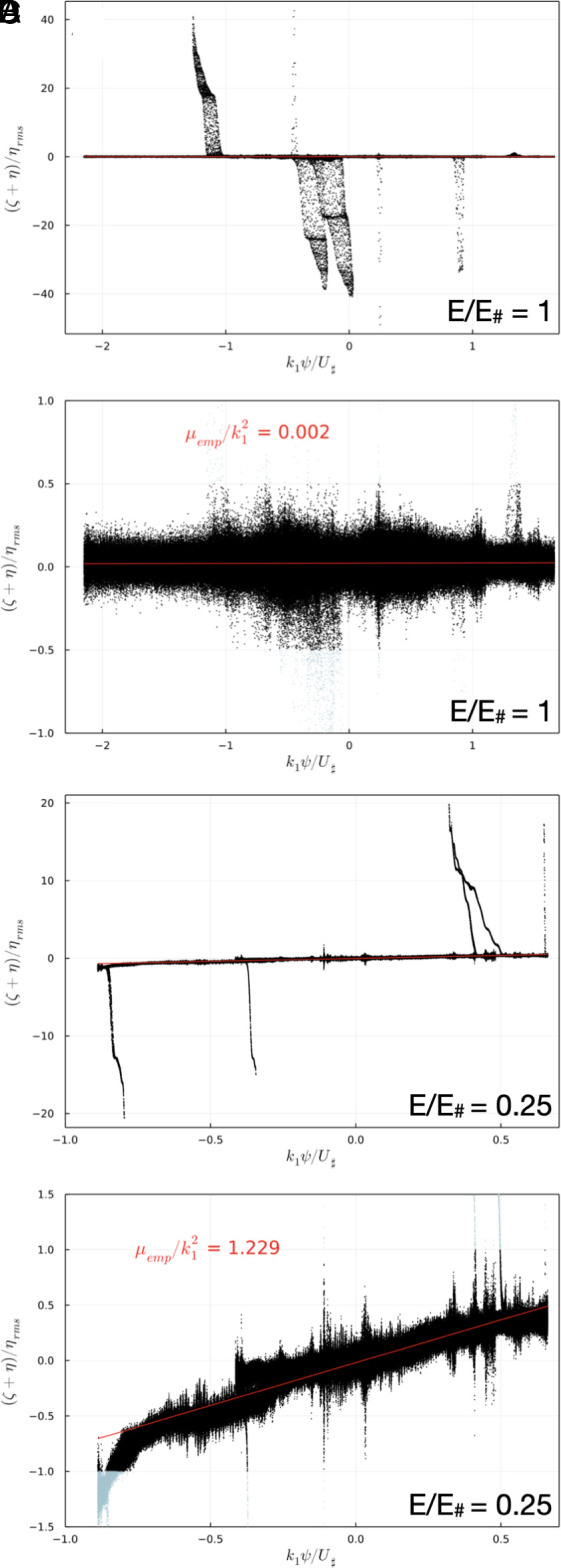
(*A*) Scatter plot between PV and ψ at final time for the run $E/E_{♯}=1$, with dt = 1,500 s. (*B*) Zoomed version of *A*. (*C*) Scatter plot between PV and ψ at final time for the run $E/E_{♯}=0.25$, with dt = 1,500 s. (*D*) Zoomed version of *B*. The red lines show the lines of best fit between PV and ψ at final time, from which empirical μ ($\mu_{\;\text{emp}}$) is derived. Light blue dots in panels *C* and *D* highlight the vortices that are excluded from the computation of the line of best fit. A flat curve, i.e., $\mu_{\;\text{emp}}=0$, is the signature of PV homogeneization (panels *A* and *C*).

To determine the empirical μ in Eq. **6** ($\mu_{\;\text{emp}}$), we compute the line of best fit in the scatter plot between PV and ψ at final time. We use a PV threshold to discard the vortices and only keep the background PV. The slope of the line of best fit corresponds to $\mu_{\;\text{emp}}$. Fig. 11 shows derivation of $\mu_{\;\text{emp}}$ for the run $E/E_{♯}=1$ and the threshold $|\left(\zeta +\eta \right)/\eta_{\;\text{rms}}|<0.5$ and the run $E/E_{♯}=0.25$ and the threshold $|\left(\zeta +\eta \right)/\eta_{\;\text{rms}}|<1$. Panels *A* and *B* show all the points and panels C and D are zoomed versions for values of $|\left(\zeta +\eta \right)/\eta_{\;\text{rms}}|\leq 2.5$. One can see that the threshold is effective at excluding the vortices and keeping the background. While PV homogeneization is apparent for $E/E_{♯}=1$, characterized by $\mu_{\;\text{emp}}=0$ (panels *A* and *C*), for $E/E_{♯}=0.25$ PV homogeneization is incomplete and $\mu_{\;\text{emp}}>0$ (panels *B* and *D*). The scatter plots for the other runs are shown in *SI Appendix*, Figs. S2–S5.

While keeping all the points in the scatter plot affects considerably the estimate of $\mu_{\;\text{emp}}$, our results are robust to threshold sensitivity (Table 2 and *SI Appendix*, Fig. S1). Hence, we picked the appropriate threshold for a given run (highlighted in green in Table 2); as the energy decreases, the threshold needs to be increased. This is because PV homogeneization is not complete at low energy runs, corresponding to μ>0, which requires to keep larger values of $|\left(\zeta +\eta \right)/\eta_{\;\text{rms}}|$ to capture the background PV.

**Table 2.** Sensitivity study

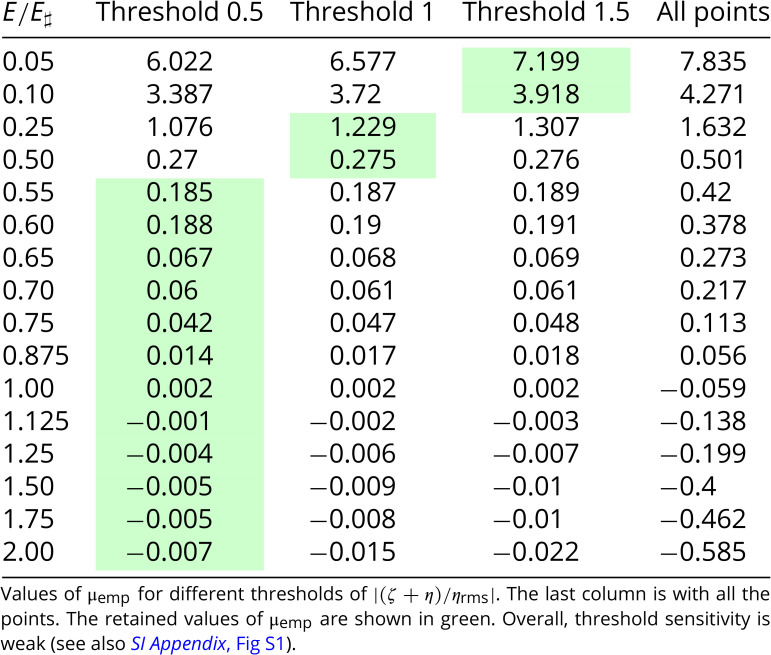

## Supplementary Material

Appendix 01 (PDF)

Movie S1. This movie shows the full 47.53 years of evolution for the run with *E/E_#_* = 0.05 for $\varsigma /\eta_{rms}$ (left panel) and $\left(\varsigma +\eta \right)/\eta_{rms}$ 67 (right panel). PV homogeneization is never complete. Topographically-locked vortices emerge in the first 4 years of evolution and remain until the end of the run. Anticyclones are trapped above depressions and cyclones above mounts.

Movie S2. This movie shows the full 47.53 years of evolution for the run with *E/E_#_* = 0.1 for $\varsigma /\eta_{rms}$ (left panel) and $\left(\varsigma +\eta \right)/\eta_{rms}$ (right panel). PV homogeneization is never complete. Topographically-locked vortices emerge in the first 4 years of evolution and remain until the end of the run. Anticyclones are trapped above depressions and cyclones above mounts.

Movie S3. This movie shows the full 47.53 years of evolution for the run with *E/E_#_* = 0.25 for $\varsigma /\eta_{rms}$ (left panel) and $\left(\varsigma +\eta \right)/\eta_{rms}$ (right panel). PV homogeneization is never complete. Topographically-locked vortices emerge in the first 4 years of evolution and remain until the end of the run. Anticyclones are trapped above depressions and cyclones above mounts.

Movie S4. This movie shows the full 47.53 years of evolution for the run with *E/E_#_* = 1 for $\varsigma /\eta_{rms}$ (left panel) and $\left(\varsigma +\eta \right)/\eta_{rms}$ (right panel). PV homogeneization is complete within a few years. Vortices orbit topographical features but are not locked to them anymore. The behavior of the flow, especially the PV, is reminiscent of classical 2D turbulence.

Movie S5. This movie shows the full 47.53 years of evolution for the run with *E/E_#_* = 2 for $\varsigma /\eta_{rms}$ (left panel) and $\left(\varsigma +\eta \right)/\eta_{rms}$ (right panel). PV homogeneization is complete within a few years. Vortices orbit topographical features but are not locked to them anymore. The behavior of the flow, especially the PV, is reminiscent of classical 2D turbulence.

Movie S6.

Movie S7.

## Data Availability

There are no data underlying this work.
