# Supplementary material for: Two-dimensional turbulence above topography: Vortices and potential vorticity homogenization
Source: Proc Natl Acad Sci U S A. 2023 Oct 23;120(44):e2308018120. doi: 10.1073/pnas.2308018120 (PMC10622930; doi:10.1073/pnas.2308018120)
Supplement: Supplementary file 1 — Appendix 01 (PDF) [file pnas.2308018120.sapp.pdf]

PNAS
www.pnas.org

Supplementary Information for

Two-dimensional turbulence above topography: vortices and potential vorticity homogenization

Lia Siegelman and William R. Young

Lia Siegelman
E-mail: lsiegelman@usd.edu

This PDF file includes:

Supplementary text
Figs. S1 to S7 (not allowed for Brief Reports)
Tables S1 to S2 (not allowed for Brief Reports)
Legends for Movies S1 to S5

Other supplementary materials for this manuscript include the following:

Movies S1 to S5

Supporting Information Text

A. Solution of the Euler-Lagrange equation

We work in an $L \times L$ doubly periodic domain with fundamental wavenumber $k_1 = 2\pi/L$. Our computations use resolution 1024×1024 and, following BH, a k^{-2} spectrum for $\eta(x, y)$. The topographic PV is represented as a Fourier series

$$\eta = \sum_{\boldsymbol{k}} \eta_{\boldsymbol{k}} \mathrm{e}^{\mathrm{i}\boldsymbol{k}\cdot\boldsymbol{x}} . \tag{1}$$

In [1]

$$\eta_{\boldsymbol{k}} = \alpha \mathrm{e}^{\mathrm{i}\phi_{\boldsymbol{k}}} k^{-3/2} , \tag{2}$$

where $k = |\boldsymbol{k}|$, $\phi_{\boldsymbol{k}}$ is a random phase and α is a normalization parameter that is adjusted to produce a specified value of

$$\eta^2_{rms} \stackrel{\text{def}}{=} \left\langle \eta^2 \right\rangle = \sum_{\boldsymbol{k}} |\eta_{\boldsymbol{k}}|^2 . \tag{3}$$

The solution of the Euler-Lagrange equation,

$$(\partial_x^2 + \partial_y^2)\psi_\star + \eta = \mu\psi_\star , \tag{4}$$

is then

$$\psi_\star(x, y, \mu) = \sum_{\boldsymbol{k}} \frac{\eta_{\boldsymbol{k}}\, \mathrm{e}^{\mathrm{i}\boldsymbol{k}\cdot\boldsymbol{x}}}{\mu + k^2} . \tag{5}$$

The special case $\mu = 0$ is the homogenized PV solution.

The energy and enstrophy associated with $\psi_\star(x, y, \mu)$ are

$$E_\star(\mu) \stackrel{\text{def}}{=} \tfrac{1}{2} \left\langle |\boldsymbol{\nabla}\psi_\star|^2 \right\rangle , \tag{6}$$

$$= \tfrac{1}{2} \sum_{\boldsymbol{k}} \frac{k^2 |\eta_{\boldsymbol{k}}|^2}{(\mu + k^2)^2} , \tag{7}$$

and

$$Q_\star(\mu) \stackrel{\text{def}}{=} \tfrac{1}{2} \left\langle (\triangle\psi_\star + \eta)^2 \right\rangle , \tag{8}$$

$$= \tfrac{1}{2} \sum_{\boldsymbol{k}} \frac{\mu^2 |\eta_{\boldsymbol{k}}|^2}{(\mu + k^2)^2} . \tag{9}$$

In figure 2 of the main text we use the results above to plot $E_\star$ as a function of μ and $Q_\star$ as a function of $E_\star$ By specifying $\mu > -k_1^2$ one can calculate E from [7] and so plot figure 2A and B.

B. Vorticity-topography correlations based ψ_{emp}

In section 5 of the main text we shows that if $E < E_\sharp$ then $\langle q\psi_\sharp \rangle > 0$. Thus on the low-energy branch there is a positive correlation between PV $q = \zeta + \eta$ and the topographic streamfunction $\psi_\sharp$. In section 4 we speculate that at very low energy levels one can empirically fit the background (outside the vortices) ψ-q with

$$\zeta + \eta \approx \mu_{\text{emp}}\psi . \tag{10}$$

In this supplementary section we show that the correlation $\langle q\psi_{\text{emp}} \rangle$ is positive provided that

$$E < \underbrace{\left\langle \tfrac{1}{2} |\boldsymbol{\nabla}\psi_{\text{emp}}|^2 \right\rangle}_{E_{\text{emp}}} \tag{11}$$

Start by observing that μ_{emp} in the range

$$0 \leq \mu_{\text{emp}} < \infty . \tag{12}$$

In th emain text we have a value of μ_{emp} suggested by diagnosis of a numerical experiment. But for the moment ignore numerical solutions and regard μ_{emp} as any positive constant. Given μ_{emp}, define $\psi_{\text{emp}}(x, y, \mu_{\text{emp}})$ as the solution of

$$\triangle\psi_{\text{emp}} + \eta = \mu_{\text{emp}}\psi_{\text{emp}} . \tag{13}$$

Given a solution of the QG equation $\psi(x, y, t)$, define $\phi(x, y, t, \mu)$ by

$$\psi(x, y, t) = \psi_{\text{emp}}(x, y, t, \mu_{\text{emp}}) + \phi(x, y, t, \mu_{\text{emp}}) . \tag{14}$$

Then the PV is

$$q = \triangle\psi + \eta\,, \qquad [15]$$
$$= \triangle\phi + \mu_{\text{emp}}\psi_{\text{emp}}\,. \qquad [16]$$

The decomposition in [14] implies that the energy, $E = \left\langle \frac{1}{2}|\nabla\psi|^2 \right\rangle$, is decomposed as

$$E = E_{\text{emp}} + \langle \nabla\psi_{\text{emp}} \cdot \nabla\phi \rangle + \left\langle \tfrac{1}{2}|\nabla\phi|^2 \right\rangle\,. \qquad [17]$$

The cross-term above is

$$\langle \nabla\psi_{\text{emp}} \cdot \nabla\phi \rangle = -\langle \psi_{\text{emp}} \triangle\phi \rangle\,, \qquad [18]$$
$$= \mu_{\text{emp}} \left\langle \psi_{\text{emp}}^2 \right\rangle - \langle \psi_{\text{emp}} q \rangle\,, \qquad [19]$$

where we have used [16] to get rid of $\triangle\phi$ in [18]. Substituting [19] into [17] and rearranging

$$\langle \psi_{\text{emp}} q \rangle = E_{\text{emp}} - E + \mu_{\text{emp}} \left\langle \psi_{\text{emp}}^2 \right\rangle + \left\langle \tfrac{1}{2}|\nabla\phi|^2 \right\rangle\,. \qquad [20]$$

In the special case $\mu_{\text{emp}} = 0$ (and thus $\psi_{\text{emp}} = \psi_\sharp$), [20] above reduces to equation [24] in the main text.

The desired result $\langle \psi_{\text{emp}} q \rangle > 0$ follows from [20] if we can show that $E_{\text{emp}} - E > 0$. In other words, provided that μ_{emp} is such that $E < E_{\text{emp}}$ it follows from Eq. (20) that $\langle \psi_{\text{emp}} q \rangle > 0$. We have verified numerically that for the runs with non-zero μ_{emp} shown in figure 7 $E_{\text{emp}} - E > 0$. Thus for these low-energy runs both $\langle q\psi_\sharp \rangle$ and $\langle q\psi_{emp} \rangle$ are positive.

C. Energy and enstrophy conservation

To a good approximation the kinetic energy is conserved throughout the evolution of the sixteen simulations. For the run $E/E_\sharp = 1$, 0.1% of the initial kinetic energy is lost in the first 90 days and another 0.1 in the remaining 47 years (Table S1). Enstrophy loss is more dramatic: 65% is lost in the first 90 days and an additional 33% in the remaining 47 years (Table S2). Note that decreasing $E/E_\sharp$ reduces the kinetic energy conservation and enstrophy loss.

$E/E_\sharp$	KE(90 days)/KE(0)	KE(1 year)/KE(0)	KE(1.6 years)/KE(0)	KE(4.3 years)/KE(0)	KE(47.53 years)/KE(0)
0.05	1.000	0.999	0.998	0.9978	0.996
0.10	1.000	0.998	0.998	0.998	0.997
0.25	0.999	0.998	0.998	0.998	0.997
0.50	0.999	0.998	0.998	0.998	0.997
0.55	0.999	0.998	0.998	0.998	0.997
0.60	0.999	0.998	0.998	0.998	0.998
0.65	0.999	0.998	0.998	0.998	0.998
0.70	0.999	0.998	0.998	0.998	0.998
0.75	0.999	0.998	0.998	0.998	0.998
0.875	0.999	0.998	0.998	0.998	0.998
1.00	0.999	0.998	0.998	0.998	0.998
1.125	0.999	0.998	0.998	0.998	0.998
1.25	0.999	0.998	0.998	0.998	0.998
1.50	0.999	0.998	0.998	0.998	0.998
1.75	0.999	0.998	0.998	0.998	0.998
2.00	0.999	0.998	0.998	0.998	0.998

Table S1. Kinetic energy (KE) at discrete times, corresponding to the snapshots in figure 3, for the solution suite.

$E/E_\sharp$	Q(90 days)/Q(0)	Q(1 year)/Q(0)	Q(1.6 years)/Q(0)	Q(4.3 years)/Q(0)	Q(47.53 years)/Q(0)
0.05	0.960	0.428	0.272	0.114	0.085
0.10	0.889	0.292	0.182	0.070	0.047
0.25	0.690	0.183	0.124	0.056	0.028
0.50	0.506	0.148	0.102	0.058	0.021
0.55	0.480	0.127	0.093	0.049	0.021
0.60	0.462	0.133	0.103	0.056	0.015
0.65	0.443	0.134	0.104	0.060	0.020
0.70	0.428	0.124	0.096	0.055	0.018
0.75	0.416	0.127	0.086	0.045	0.016
0.875	0.381	0.116	0.087	0.056	0.017
1.00	0.354	0.115	0.090	0.052	0.018
1.125	0.331	0.106	0.087	0.052	0.017
1.25	0.317	0.100	0.078	0.051	0.017
1.50	0.297	0.113	0.096	0.065	0.026
1.75	0.270	0.102	0.079	0.053	0.022
2.00	0.257	0.099	0.079	0.058	0.025

Table S2. Enstrophy (Q) at discrete times, corresponding to the snapshots in figure 3, for the solution suite.

D. Supplementary figures in support of the main text and the material and methods

The following figures are referred to in the main text and the material and methods.

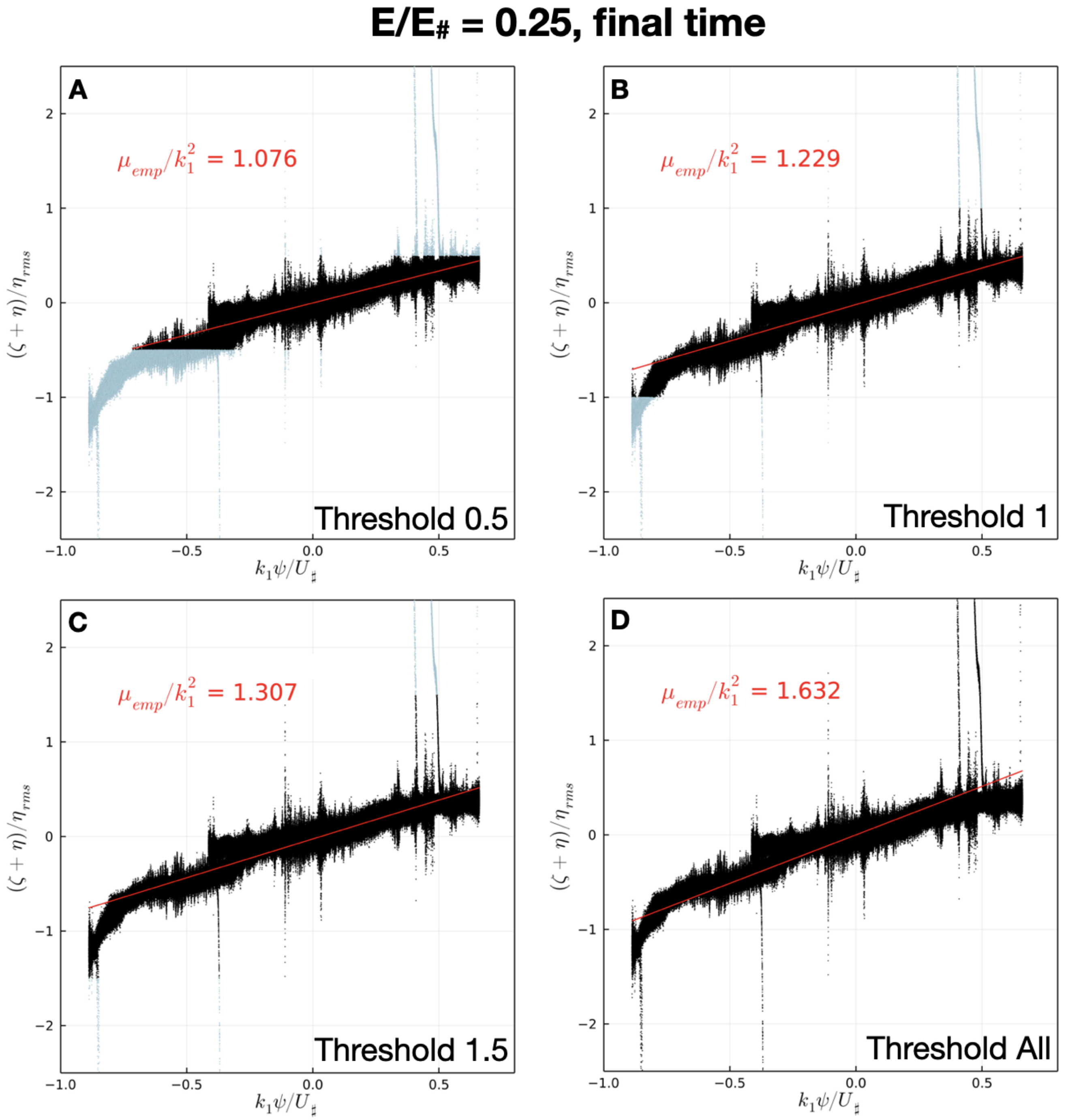

Fig. S1. Impact of the threshold on μ_{emp} for the run with $E/E_\sharp = 0.25$. A. Points such that $|(\zeta+\eta)/\eta_{\mathrm{rms}}| > 0.5$ are excluded. B. Points such that $|(\zeta+\eta)/\eta_{\mathrm{rms}}| > 1$ are excluded. C. Points such that $|(\zeta+\eta)/\eta_{\mathrm{rms}}| > 1.5$ are excluded. These points are shown in light blue in panels A-C. D. All points are conserved. We decided to use threshold 1 (panel B). In panel A, part of the background PV is removed, hence the threshold is too conservative. In panel D, the strong vortices are conserved and influence the derivation of μ_{emp}. However, the thresholds in panels B and C do a good job of removing most of the vortices and keeping the background. The difference on μ_{emp}/k_1^2 is less than 0.1, or 6%, highlighting the weak sensitivity of our results to the threshold's choice.

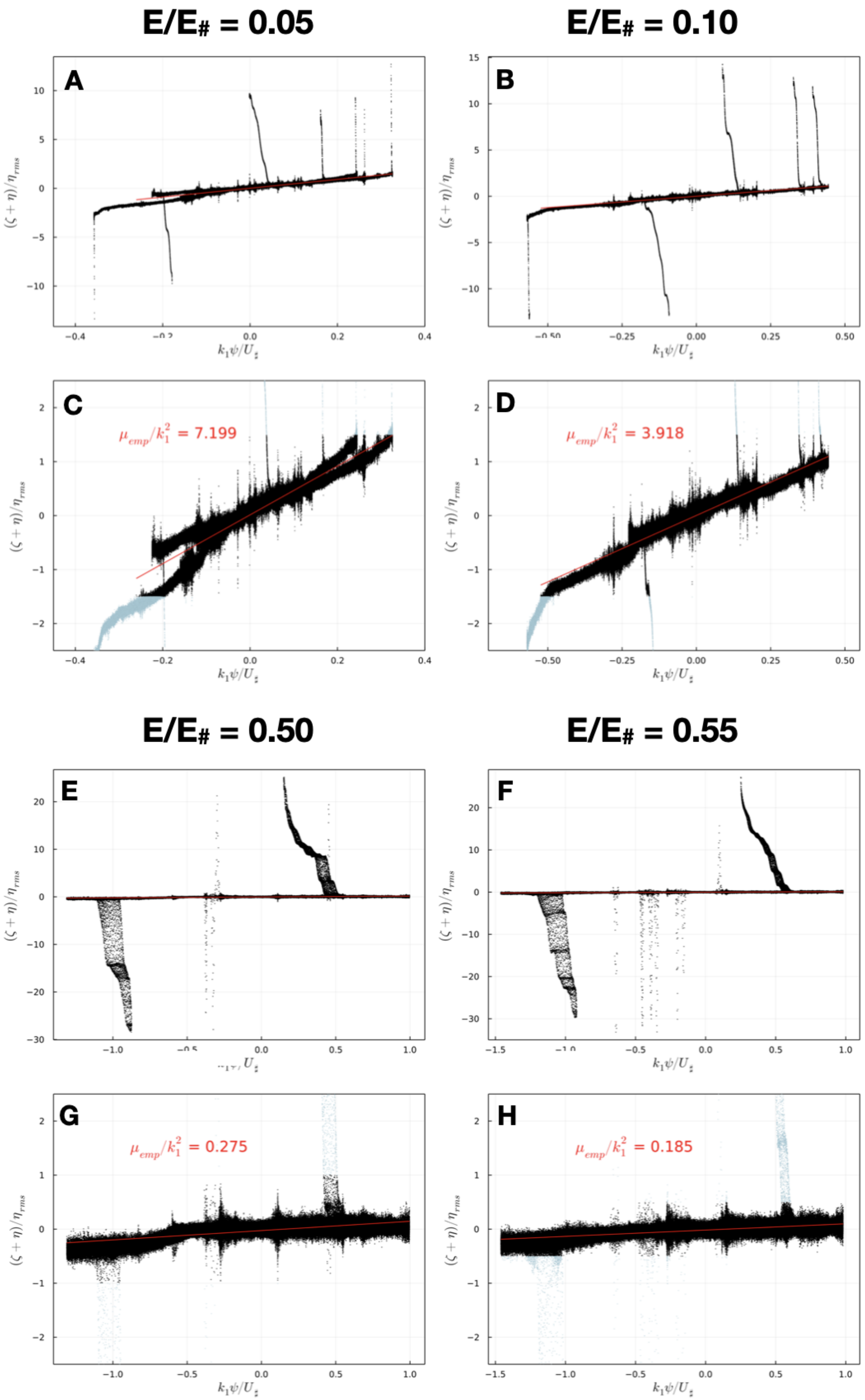

Fig. S2. Scatter plots between PV and ψ used to determine μ_{emp}. Same as figure 11 in the methods but for $E/E_\sharp = 0.05$ (panels A and C), $E/E_\sharp = 0.10$ (panels B and D), $E/E_\sharp = 0.50$ (panels E and G), $E/E_\sharp = 0.55$ (panels F and H). Note that the distribution of ψ is asymmetric due to vortex nucleation, which randomly produces cyclones or anticyclones. Cyclones are associated with positive PV and negative ψ and anticyclones are associated with negative PV and positive ψ.

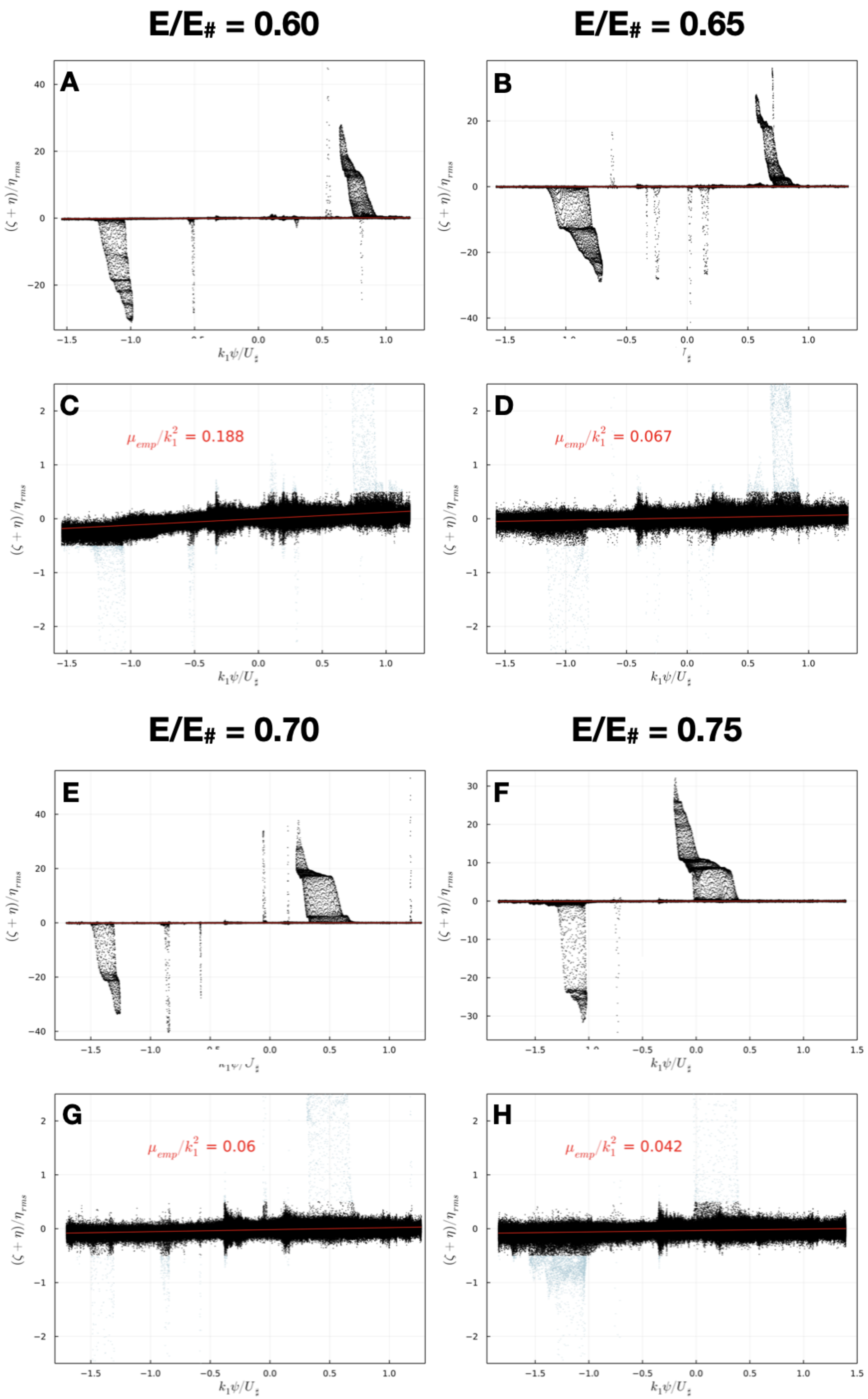

Fig. S3. Scatter plots between PV and ψ used to determine $\mu_{\rm emp}$. Same as figure X in the methods but for $E/E_\sharp = 0.60$ (panels A and C), $E/E_\sharp = 0.65$ (panels B and D), $E/E_\sharp = 0.70$ (panels E and G), $E/E_\sharp = 0.75$ (panels F and H).

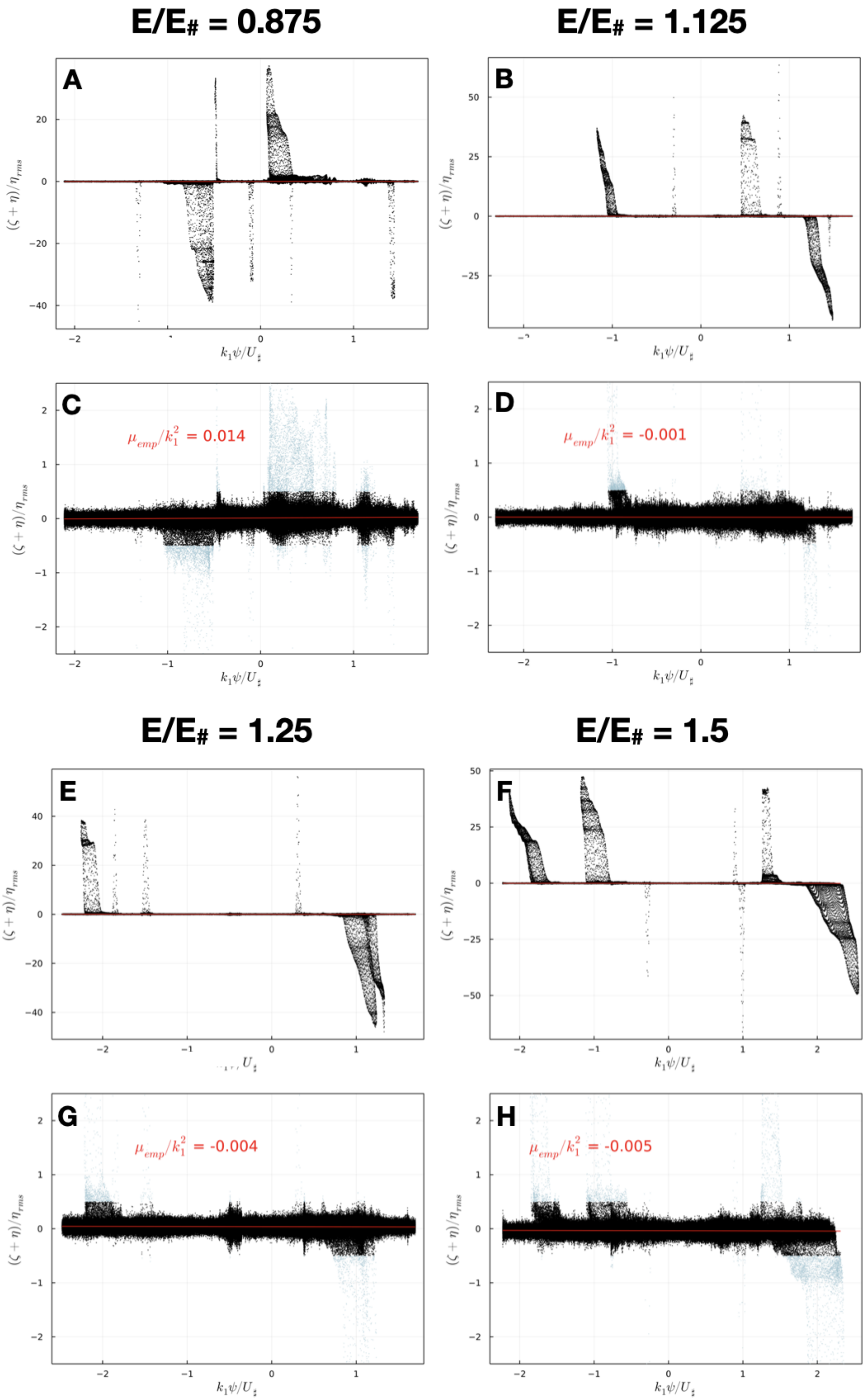

Fig. S4. Scatter plots between PV and ψ used to determine μ_{emp}. Same as figure X in the methods but for $E/E_\sharp = 0.875$ (panels A and C), $E/E_\sharp = 1.125$ (panels B and D), $E/E_\sharp = 1.25$ (panels E and G), $E/E_\sharp = 1.5$ (panels F and H).

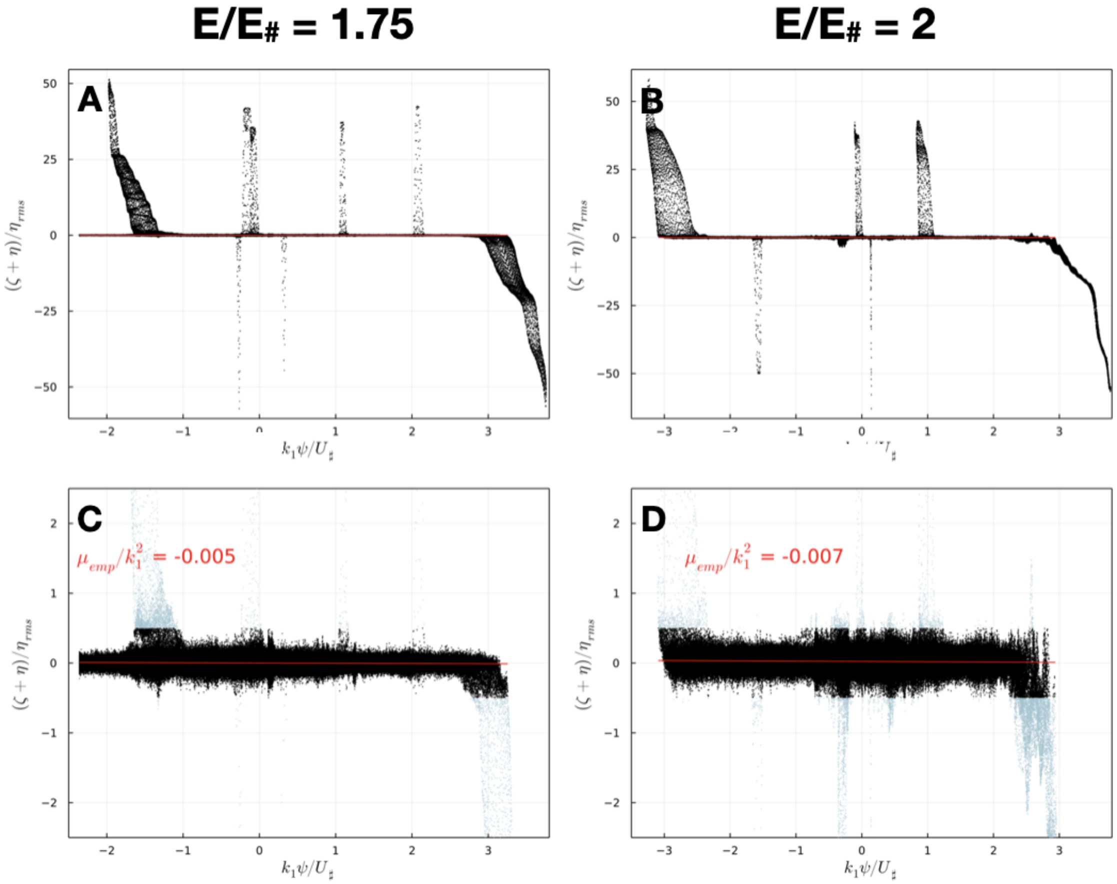

Fig. S5. Scatter plots between PV and ψ used to determine μ_{emp}. Same as figure X in the methods but for $E/E_\sharp = 1.75$ (panels A and C), $E/E_\sharp = 2$ (panels B and D).

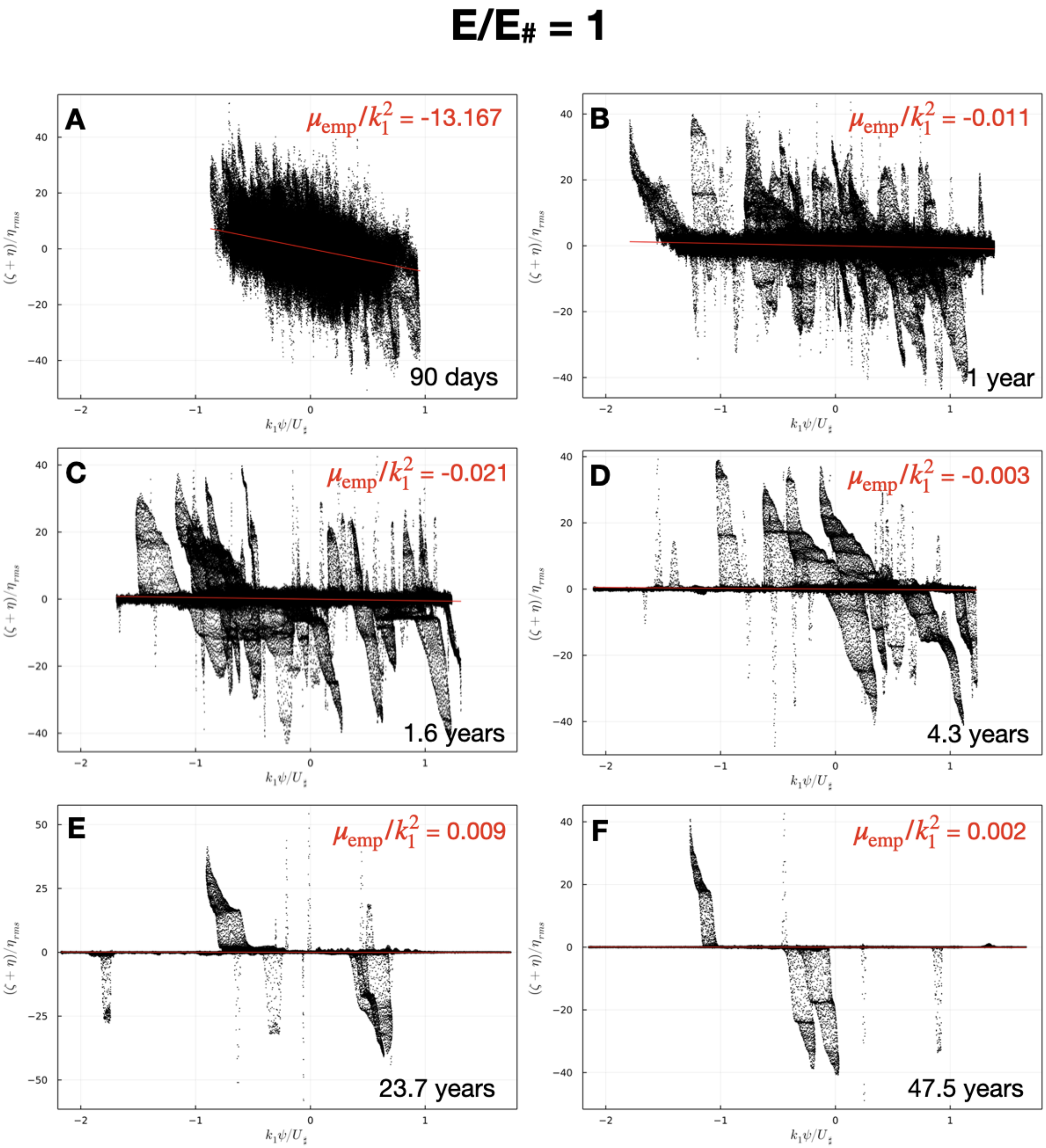

Fig. S6. Time evolution of $\mu_{\rm emp}$ for the run with $E/E_\sharp = 1$. $\mu_{\rm emp}/k_1^2$ remains smaller than 0.01 from ~ 4.3 years, indicating that PV homogeneization is complete at that time. The vortices correspond to the overshooting positive (cyclones) and negative (anticyclones) dots on the y-axis. Vortex nucleation and merger is apparent across time. For instance, at 47.5 years in panel F, there remains two cyclones and 4 anticyclones.

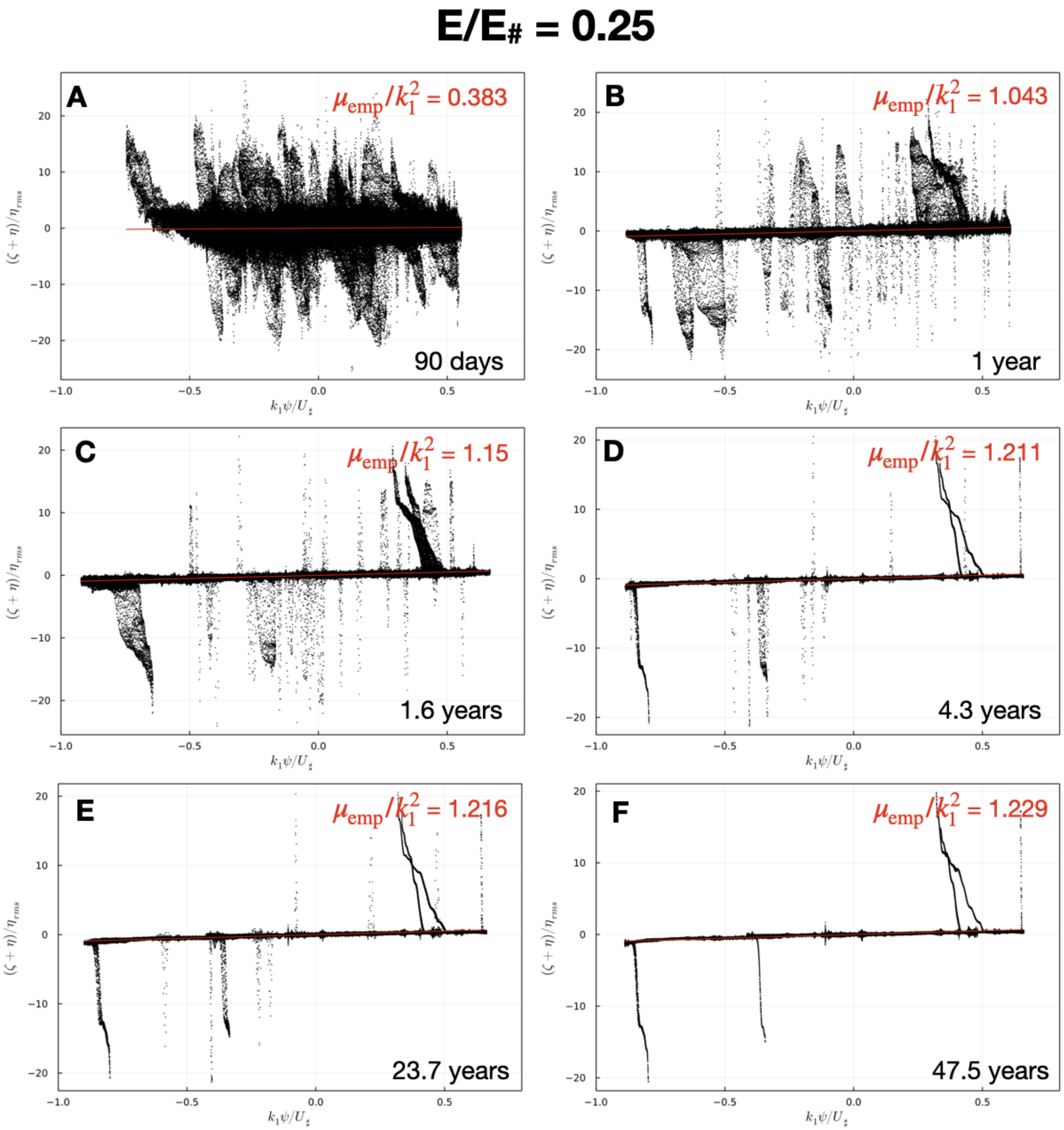

Fig. S7. Time evolution of $\mu_{\rm emp}$ for the run with $E/E_\sharp = 0.25$. $\mu_{\rm emp}$ remains constant from ~ 4.3 years of evolution. This duration is observed for all runs. Vortices are apparent like in Fig. S6.

E. Movie legends

Movie S1. This movie shows the full 47.53 years of evolution for the run with $E/E_\sharp = 0.05$ for ζ/η_{rms} (left panel) and $(\zeta+\eta)/\eta_{rms}$ (right panel). PV homogeneization is never complete. Topographically-locked vortices emerge in the first 4 years of evolution and remain until the end of the run. Anticyclones are trapped above depressions and cyclones above mounts.

Movie S2. This movie shows the full 47.53 years of evolution for the run with $E/E_\sharp = 0.1$ for ζ/η_{rms} (left panel) and $(\zeta+\eta)/\eta_{rms}$ (right panel). PV homogeneization is never complete. Topographically-locked vortices emerge in the first 4 years of evolution and remain until the end of the run. Anticyclones are trapped above depressions and cyclones above mounts.

Movie S3. This movie shows the full 47.53 years of evolution for the run with $E/E_\sharp = 0.25$ for ζ/η_{rms} (left panel) and $(\zeta+\eta)/\eta_{rms}$ (right panel). PV homogeneization is never complete. Topographically-locked vortices emerge in the first 4 years of evolution and remain until the end of the run. Anticyclones are trapped above depressions and cyclones above mounts.

Movie S4. This movie shows the full 47.53 years of evolution for the run with $E/E_\sharp = 1$ for ζ/η_{rms} (left panel) and $(\zeta+\eta)/\eta_{rms}$ (right panel). PV homogeneization is complete within a few years. Vortices orbit topographical features but are not locked to them anymore. The behavior of the flow, especially the PV, is reminiscent of classical 2D turbulence.

Movie S5. This movie shows the full 47.53 years of evolution for the run with $E/E_\sharp = 2$ for ζ/η_{rms} (left panel) and $(\zeta+\eta)/\eta_{rms}$ (right panel). PV homogeneization is complete within a few years. Vortices orbit topographical features but are not locked to them anymore. The behavior of the flow, especially the PV, is reminiscent of classical 2D turbulence.
